# Study on the Comprehensive Phytochemicals and the Anti-Ulcerative Colitis Effect of *Saussurea pulchella*

**DOI:** 10.3390/molecules28041526

**Published:** 2023-02-04

**Authors:** Yunhe Liu, Caixia Wang, Junzhe Wu, Luying Tan, Peng Gao, Sinuo Wu, Daohao Tang, Qianyun Wang, Cuizhu Wang, Pingya Li, Jinping Liu

**Affiliations:** 1 School of Pharmaceutical Sciences, Jilin University, Changchun 130021, China; 2 Research Center of Natural Drugs, Jilin University, Changchun 130021, China

**Keywords:** *Saussurea pulchella*, chemical composition, ulcerative colitis, metabolomics, network pharmacology

## Abstract

Background: *Saussurea pulchella (SP)* is a traditional medicinal plant that is widely used in folk medicine because of its diverse biological activities, particularly its anti-inflammatory effects. However, the alleviation effect of SP on ulcerative colitis (UC) has not yet been realized. Purpose: To investigate the chemical composition and therapeutic effect of SP extract against UC. Methods: First, qualitative and quantitative analysis of SP 75% ethanol extract was performed by UPLC-Q/TOF-MS. Second, a dextran sodium sulfate (DSS) model of UC mice was developed to study the effects of SP on the symptoms, inflammatory factors, oxidative stress indexes and colon histopathology. Third, an integration of network pharmacology with metabolomics was performed to investigate the key metabolites, biological targets and metabolisms closely related to the effect of SP. Results: From the SP ethanol extract, 149 compounds were identified qualitatively and 20 were determined quantitatively. The SP could dose-dependently decrease the DAI score, spleen coefficient and the levels of TNF-α, IL-6, iNOS, MPO and MDA; increase the colon length, GSH level and SOD activity; and protect the intestinal barrier in the UC mice. Moreover, 10 metabolite biomarkers,18 targets and 5 metabolisms were found to play crucial roles in the treatment of UC with SP. Conclusions: SP 75% ethanol extract could effectively alleviate the progression of UC and, therefore, could be classified as a novel natural treatment for UC.

## 1. Introduction

Ulcerative colitis (UC), an inflammatory disease, has increasing prevalence worldwide [1]. The typical symptoms of UC include bloody diarrhea, tenesmus and abdominal pain [2]. At present, drug intervention is the main method to treat UC. The main first-line drugs are aminosalicylic acids, glucocorticoids, immunomodulators and biological drugs. However, there are also some disadvantages, such as dose-dependent toxicity, drug dependence, irreversible complications or high cost [3]. The research and development of safer, more effective and more economical drugs has become a hotspot in research. With the tide of global natural medicines, products that have the effect of clearing heat and removing dampness, such as *Coptis chinensis*, *Glycyrrhiza uralensis*, *Croton crassifolius* and Shaoyao Decoction, have been used to treat UC in clinic. To our satisfaction, the curative effects were unique and remarkable, and the toxicity was also low [4,5,6,7,8].

*Saussurea pulchella* (SP), with the functions of dispelling wind, clearing heat, removing dampness and relieving pain [9], is widely distributed in northeastern Asia, particularly in China, Japan, Korea and Russia [10,11,12]. In Korea, SP had been used as folk medicine for the treatment of inflammation, hypertension, hepatitis and arthritis [12]. In Russia, SP has been used to treat diarrhea [13]. While, in our country, it has been widely used in folk medicine for treating rheumatoid arthritis, hepatitis, diarrhea and other diseases [14,15]. Modern pharmacological research has also demonstrated that the ethanol extract of SP and the sesquiterpenes from SP both exerted obvious anti-inflammatory effects [12,16,17]. It is noteworthly that, *Saussurea lappa*, which is in the same family and has the same genus as SP, has also been reported to have the anti-ulcecrative effect. For example, the sesquiterpenes isolated from *Saussurea lappa* methanol extract could alleviate HCl/ethanol-induced gastric mucosal lesions in gastric ulcer rats [18]. In gastric ulceration rats and duodenal ulceration rats, the ethyl acetate extract of *Saussurea lappa* showed good anti-ulcer activity [19]. However, there has been no report about the effect of SP on ulcerative colitis. It has been reported that SP contains a variety of secondary metabolites, such as phenylpropanoids, flavonoids and terpenoids [20]. However, the chemical composition of SP is still not clear; namely, there is no literature report on the comprehensive phytochemicals analysis or the quantitative assay of the main chemical components of SP.

This work is intended to investigate the chemical components and anti-ulcerative colitis activity of SP. Firstly, by using UPLC-Q/TOF-MS, the collective understanding of the chemical components of SP was refined based on the UNIFI platform, and the content assay of the main phenylpropanoids and flavonoids in SP was also performed. Secondly, a dextran sulfate sodium (DSS)-induced UC mouse model was established to evaluate the anti-ulcerative colitis activity of SP by examining the biochemical indicators, disease activity index, histopathological changes, etc. Thirdly, to identify the key metabolite biomarkers, targets and metabolisms linked with the effect of SP, an integrated analysis of metabolomics and network pharmacology was carried out. This research is conducive to illustrating the chemical components of SP and to providing a theoretical basis for expanding the application of SP. In addition, the study could also provide a potential natural medicine with good anti-ulcerative colitis activity.

## 2. Results

### 2.1. Comprehensive Phytochemical Analysis

#### 2.1.1. Qualitative Analysis

**Figure 1:**
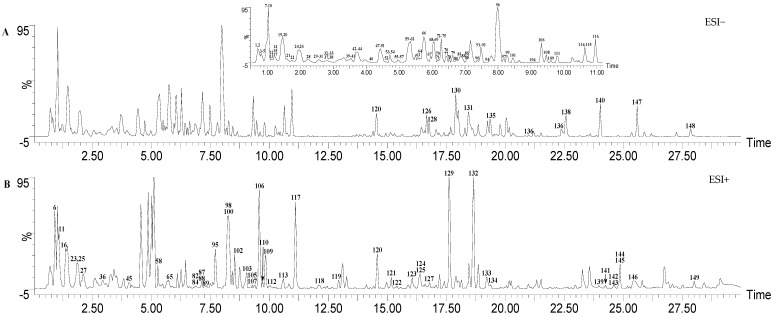
The base peak intensity (BPI) chromatograms of SP in ESI− and ESI+ modes.

The base peak intensity (BPI) chromatograms of the SP 75% ethanol extract are shown in Figure 1. A total of 149 components were identified or tentatively identified (Table 1). Among them, 35 components were identified through a comparison with the reference substances, while other components were preliminarily identified through accurate molecular weight and typical mass fragment analysis. It is also worth mentioning that 139 of the 149 components were identified from SP for the first time. The identification of these phytochemicals highlights the structural diversity of secondary metabolites in SP.

**Figure 2:**
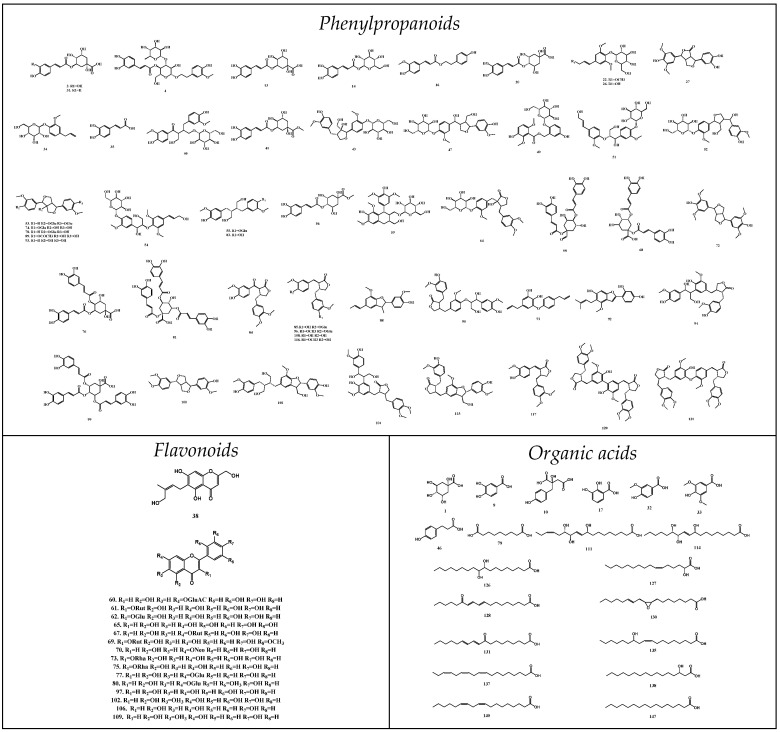

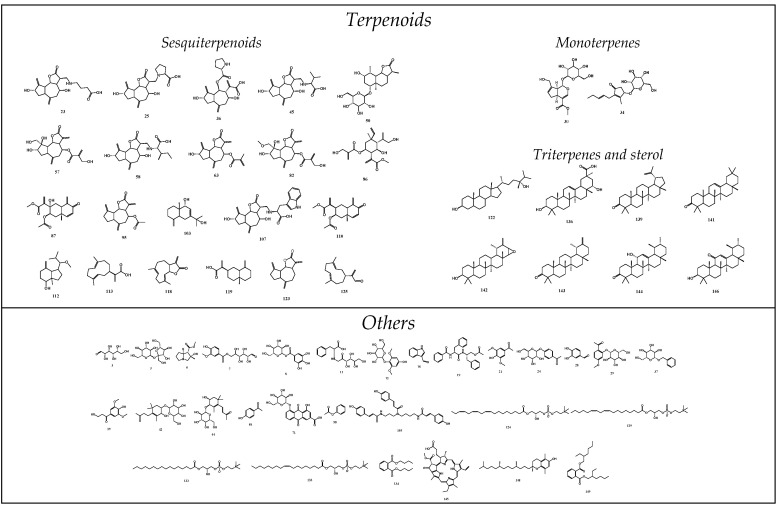
Chemical structures of components identified in SP.

According to the types of chemical structure, these identified compounds could be divided into phenylpropanoids, flavonoids, terpenoids, organic acids and other types. The structures are listed in Figure 2.

#### 2.1.2. Quantitative Analysis

*Methodological verification* The RSDs of accuracy and precision, displayed in Table S1, were all less than 3.0%. The average recoveries of 20 compounds were all more than 95%. The LOD, LOQ and linear relationships are presented in Table 1. The detection and quantitation limits of the 20 components were within the appropriate ranges, and the standard curves exhibited good linearity over the corresponding ranges. The results showed that the method could be used for the quantitative assay of the main polyphenols of SP ethanol extract.

*Quantitative Analysis results* The contents of all of the compounds are summarized in Table 2. The results showed that 20 polyphenols accounted for 33.2% of the ethanol extract of SP. Among them, the chemical components with high contents were narcisin (6.94%), rutin (6.86%), arctiin (5.42%), chlorogenic acid (4.60%), apigenin (4.10%), 1,4-dicaffeoylquinic acid (2.04%) and pinoresinol (1.12%).

### 2.2. Alleviated Ulcerative Colitis Activity

#### 2.2.1. Body Weights, Clinical Signs Observations and DAI

**Figure 3:**
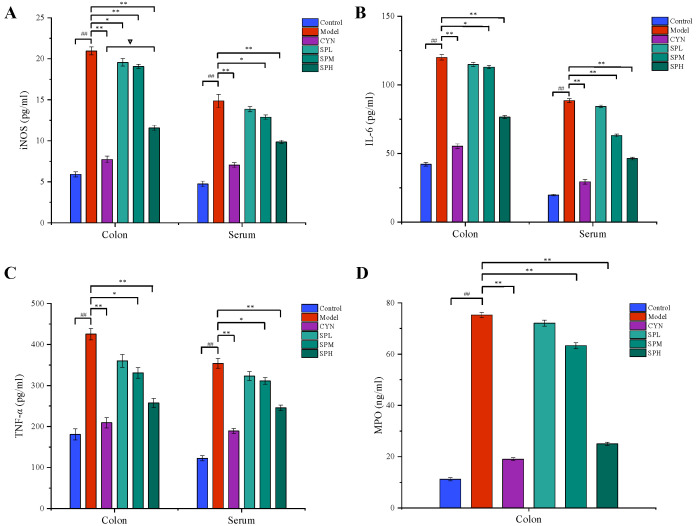
Effect of SP on (**A**) body weight, (**B**) disease activity index, (**C**) colon length and (**D**) spleen coefficient. (Data are expressed as the means ± S.E.M., *n* = 10). Compared with control group, ^##^ *p* < 0.01; compared with model group, * *p* < 0.05, ** *p* < 0.01; compared with CYN group, ∇ *p* > 0.05).

Throughout the experiment, the mice in the control group had normal weight growth, clinical signs and DAI. In contrast, the mice in the model group developed obvious anorexia and weight loss. As for the mice intervened with CNY or SP, the weight loss and clinical signs were alleviated to various degrees. From the fifth day of administration, SP dose-dependently reduced the DSS-mediated increase in the DAI scores during the disease progression compared with the model mice. By the seventh day, the UC model mice became more symptomatic with the increasing DSS induction time, as evidenced by loose stools, blood in the stool and the DAI scores. On the tenth day, the weights in all of the administration groups, except the SPL group, were higher than those in the model group (*p* < 0.05, Figure 3A), and the DAI scores in all of the administration groups, except the SPL group, were lower than those in the model group (*p* < 0.01, Figure 3B).

#### 2.2.2. Colon Length and Spleen Coefficient

As shown in Figure 3C, the colon of the model group mice was significantly shorter than that of the control group, indicating that the colon tissue had been damaged (*p* < 0.01). Colon damage could be reduced with the oral administration of SP or CYN. Compared to the model group, a significant increase (*p* < 0.01) in colon length was observed in the CYN, SPM and SPH groups, and high doses of SP provided a similar effect to CYN (*p* > 0.05). Compared to the control group, the spleen coefficient increased significantly in the model group, indicating that the UC mice exhibited inflammatory responses. In contrast, after CYN or three dosages of SP, the spleen coefficients were significantly decreased (*p* < 0.01). The above results are shown in Figure 3D. The above results showed that both SP and the positive drug could decrease the inflammatory responses in UC mice.

#### 2.2.3. Measurement of Cytokines and MPO Contents

**Figure 4:**
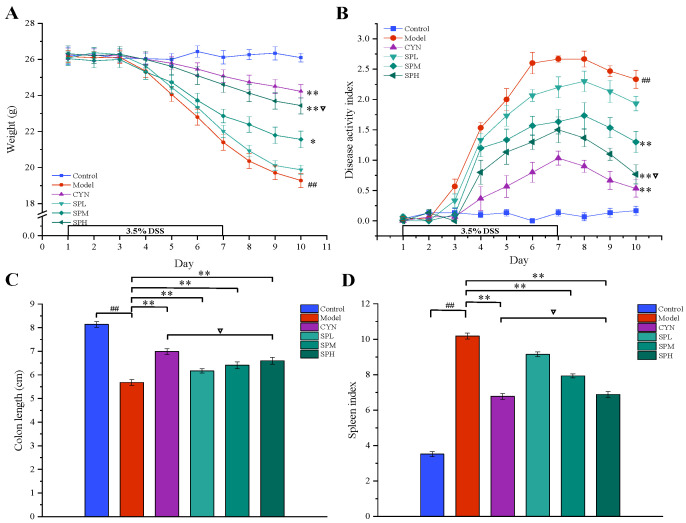
Effect of SP on (**A**) iNOS, (**B**) IL-6, (**C**) TNF-*α* and (**D**) MPO. (Data are expressed as the means ± S.E.M., *n* = 10). Compared with control group, ^##^ *p* < 0.01; compared with model group, ** *p* < 0.01; compared with CYN group, ∇ *p* > 0.05).

As shown in Figure 4, the levels of TNF-α, IL-6 and iNOS in the serum or colon, and the level of MPO in the colon, were all considerably higher in the model group compared to the control group. While being treated with CYN and SP, the levels of the above cytokines all significantly decreased compared with the model group. In addition, in terms of modulating TNF-α and MPO, the SP at a high dose showed similar effects to CYN, the positive control drug.

#### 2.2.4. Measurement of Oxidative Stress Indexes Levels

**Figure 5:**
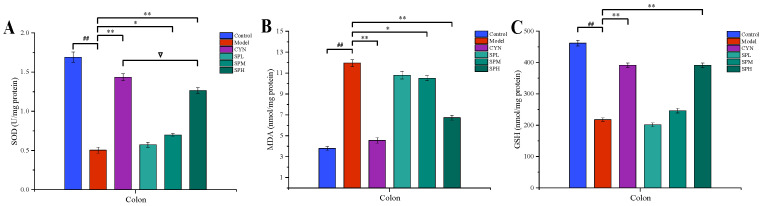
Effect of SP on (**A**) SOD, (**B**) MDA and (**C**) GSH. (Data are expressed as the means ± S.E.M., *n* = 10). Compared with control group, ^##^ *p* < 0.01; compared with model group, * *p* < 0.05, ** *p* < 0.01; compared with CYN group, ∇ *p* > 0.05).

Lipid peroxidation is associated with ulcerative colitis due to oxidative damage. The activated free radicals will deplete the antioxidant level in the colon and aggravate ulcerative colitis. As demonstrated in Figure 5, compared with the control group, the GSH and SOD levels of the model group mice significantly decreased, while the MDA level significantly increased. However, compared with the model group, after the intervention of CYN or SPH, the levels of GSH and SOD in the mice were significantly increased, while the level of MDA was significantly reduced.

#### 2.2.5. Histopathology

**Figure 6:**
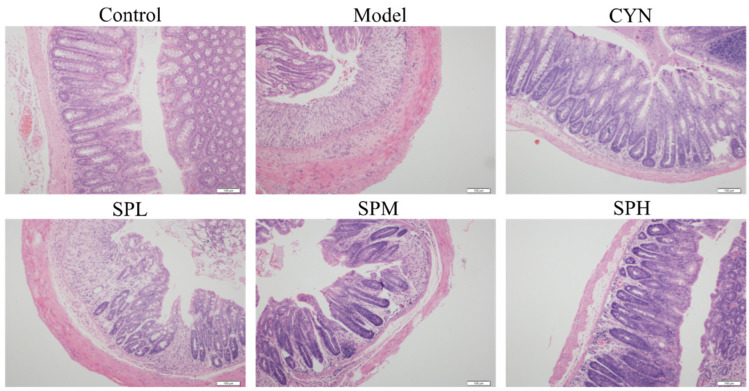
The typical H&E staining photos of the colon section.

The typical H&E staining photos are list in Figure 6. In the control group, the normal whole colonic structure and mucosal epithelium was visible. Severe mucosal damage and edema in the submucosal region and goblet cell were found in the model group. Compared with the model group, the inflammatory cell infiltration in the SP and CYN groups was decreased, the epithelial damage was recovered, and the colonic tissues were relatively complete, indicating that the inflammatory symptoms of the colonic tissue in each group were alleviated to various degrees after CYN or SP intervention.

#### 2.2.6. Transmission Electron Microscopy Analysis

**Figure 7:**
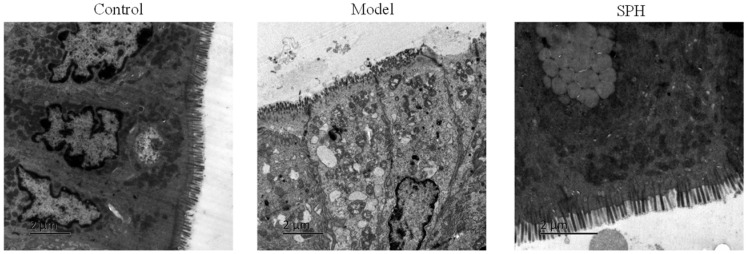
The transmission electron microscopy photos in colon section.

To confirm the effect of SP on the intestinal microvilli, transmission electron microscopy analysis was performed. The results are shown in Figure 7. In the control group, the villi of the colonic epithelial cells were neatly arranged and fully formed. However, various degrees of villous shedding and disorder were observed in the model group. Meanwhile, vacuolar degeneration was seen in mitochondria. For the SPH group, the villi arranged neatly without obvious shedding and organelle morphology is intact and normal.

### 2.3. Metabolomics

#### 2.3.1. Validation and Determination

The m/z-RT pairs in the ESI+ mode and ESI− mode included 132.0865-0.67, 274.2741-12.64, 362.3267-12.98, 104.1092-17.08, 496.3401-17.58; and 286.8602-0.59, 191.0191-0.78, 329.2319-10.40, 233.1547-16.68, 452.2766-17.94, respectively. The RSDs of the peak intensity and RT for the system stability, precision, reproducibility and sample stability were calculated and are listed in Table S2; they were all less than 3.0%. It was indicated that the established method with good precision, reproducibility and stability could be applied to assay the serum and colon samples. The detected representative base peak intensity (BPI) chromatograms of the serum and colon samples are shown in Figure S1.

#### 2.3.2. Multivariate Statistical Analyses of Serum and Colon Metabolomics

**Figure 8:**
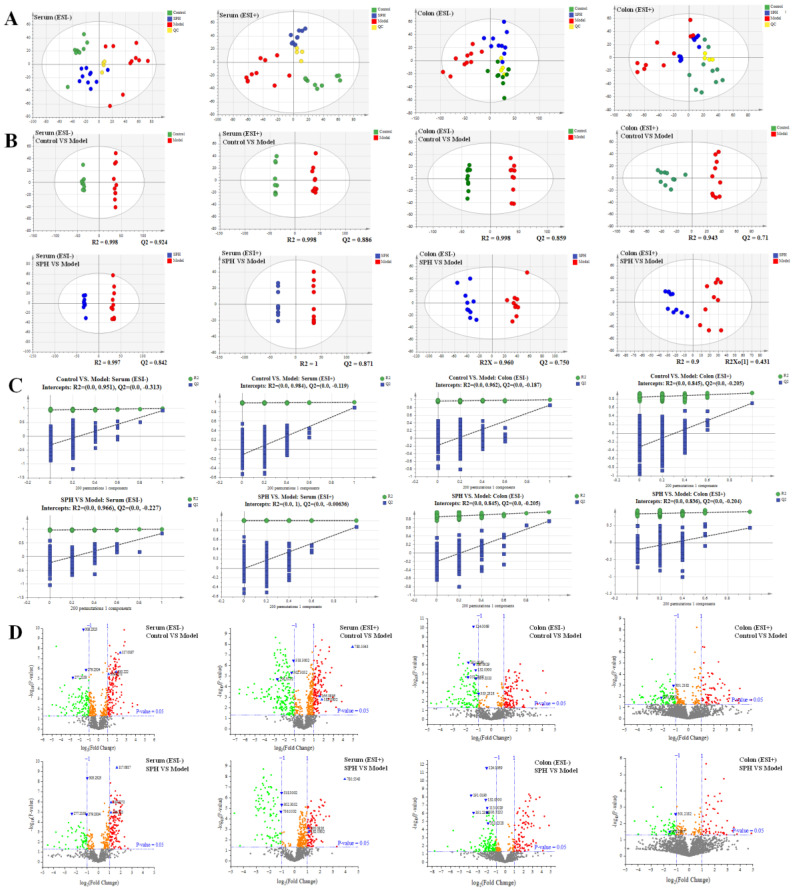
The PCA score (**A**), OPLS-DA score (**B**), permutations test (**C**) and volcano plots (**D**) of serum and colon metabolic profiling.

**Figure 9:**
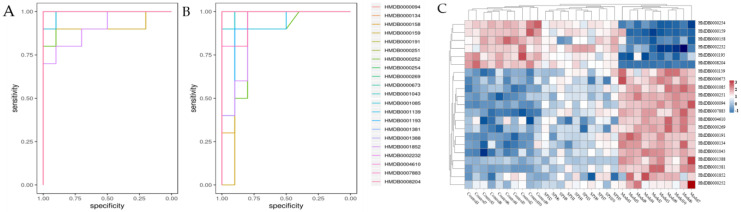
The predictive receiver operating characteristic (ROC) curves (**A**: between control group and model group, **B**: between model group and SPH group) and the heatmap (**C**) of the identified potential biomarkers in each group.

The metabolomic study was performed in both the ESI+ and in ESI− modes. A satisfactory level of system stability was also shown by the clustered QC samples in the PCA results (Figure 8A). The tested serum or colon samples from the control, model or SPH groups were clustered, respectively. The samples from the three groups were located in different regions, indicating that the metabolic disturbances in the three groups were differential. In order to achieve maximal separation between two groups, the OPLS-DA models were then established (Figure 8B). The separation between the control group and model group, or between the SPH group and model group, were achieved with satisfactory R2Y values and Q2 values. Moreover, the permutation test (Figure 8C) also showed that all of the Q2-values to the left were lower than the original points to the right, indicating that the OPLS-DA models were valid. Volcano maps (Figure 8D) were further performed to screen the differentiated metabolites. As a result, a total of 21 metabolites were identified and given the red color. Moreover, the generated ROC curves (Figure 9A,B) analyzed the above 21 metabolites, and the AUC values (all greater than 0.8) and *p* values (all less than 0.01) are listed in Table S3. All of them have the potential to be used as UC diagnostic biomarkers, according to the ROC analysis between the model group and the control group. The analysis of the ROC curves between the model and SPH groups showed that these metabolites contributed to the effects of SPH in UC treatment.

#### 2.3.3. Biomarkers Screening and Pathway Enrichment

As potential biomarkers, 21 endogenous metabolites were identified (Table 3). After that, these potential biomarkers from different groups were visualized and mapped on the heat map (Figure 9C). From blue to red, the colors indicated increasing abundance of the metabolites.

The MetaboAnalyst analysis revealed that the 21 potential biomarkers were mainly associated with 11 potential metabolisms with impact values above 0.10 (Table 4).

### 2.4. Network Pharmacology

The intersection of 1532 SP-related targets and 4920 UC-related targets provided a total of 373 core targets. Inflammatory factors, such as IL-6, TNF, NOS2 and MPO, determined in the study of the anti-UC activity, are also included in these targets. Among the various targets, enzymes (137 species) accounted for the greatest fraction (36.73%), followed by kinases (16.89%).

**Figure 10:**
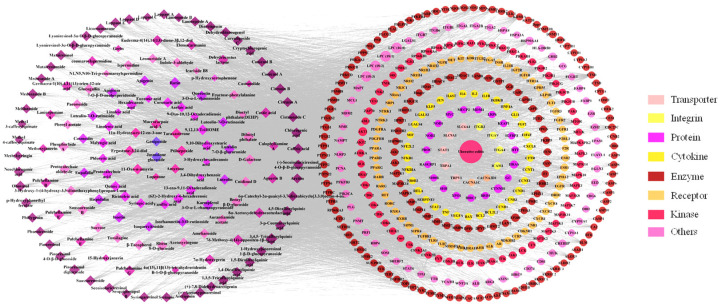
The network of SP-core targets and PPI network.

Next, the interactions of 149 compounds on 373 core targets were examined, and the SP-core targets network was built, as shown in Figure 10, which illustrated a network with 535 nodes and 11,387 edges. On one hand, 116 of the components’ degrees were greater than the average degree, which is 65. Among these 116 components, there were 17 components that had been quantified determined. On the other hand, the degrees of the phenylpropanoids and flavonoids, being 75 and 74, respectively, were greater than the other structure type’s component degrees.

Based on the aforementioned topology analysis, the components with high degree values (indicating that more targets were related) might be regarded as potential active components. In addition, the PPI network was also developed to identify potential targets for SP against UC.

### 2.5. Integrated Analysis Involving Metabolomics and Network Pharmacology

**Figure 11:**
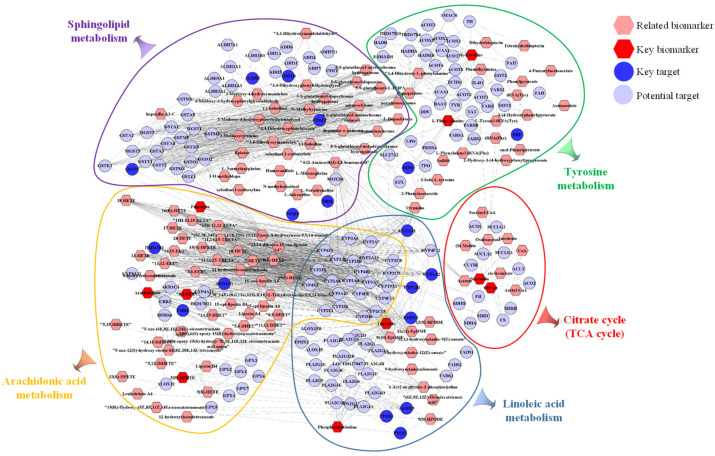
The network of “biomarkers-targets-pathways.

**Figure 12:**
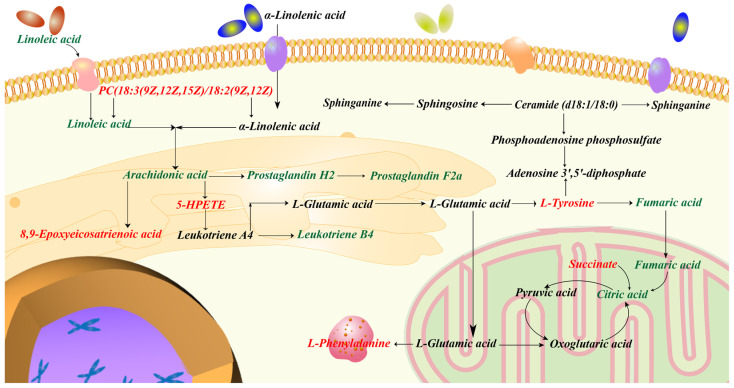
Metabolic network including key biomarkers and their metabolic sites.

The integrated analysis was performed based on the 373 potential targets obtained from the network pharmacology and the 21 potential biomarkers identified from the metabolomics in order to further confirm the key targets, biomarkers and pathways. The “biomarkers-targets-pathways” network was then constructed (Figure 11). Through matching analysis, there were ten biomarkers (succinate, L-phenylalanine, L-tyrosine, fumarate, PC(18:3/18:2), citrate, arachidonate, linoleic acid, 5(S)-HpETE, 8,9-EET), 18 targets (ADH1B, AKR1C1, ALDH2, ALOX5, CBR1, COMT, CYP1A1, CYP1A2, CYP1B1, DBH, EPHX1, GSTP1, HSD11B2, MIF, MPO, PNMT, PTGS1, PTGS2) and five pathways (arachidonic acid metabolism, citrate cycle, linoleic acid metabolism, sphingolipid metabolism and tyrosine metabolism) that were closely connected in the constructed network. In addition, the metabolic network, including these ten key biomarkers and their metabolic sites, is shown in Figure 12.

## 3. Discussion

In this study, the chemical composition and pharmacological effect of relieving ulcerative colitis with SP 75% ethanol extract were investigated for the first time. It sheds fresh light on the medical significance of SP as a viable candidate for alleviating UC symptoms.

Both the qualitative and quantitative characteristics of SP extract were determined by UPLC-Q/TOF-MS. A total of 149 components were identified. It was reported that both the phenylpropanoids and flavonoids have anti-UC effects [104,105,106,107]. Therefore, we quantitatively assayed the twelve phenylpropanoids and eight flavonoids in the SP extract. In addition, a total of 116 components (17 of them were quantified) with degrees greater than the average degree were screened as the potential active components in network pharmacology. Interestingly, the degrees of the phenylpropanoids and flavonoids were higher than the other structure types, which suggested that these two kinds of substances contributed the most to the pharmacological activity of SP. The above chemical composition research results provided the material basis for the pharmacological activity of SP.

As DSS consumption could damage the intestinal epithelium chemically, expose the lamina propria to lumen antigens and intestinal bacteria, and trigger an inflammatory and immunological response in the gut [108], an experimental model of UC in mice was established, and induced by using DSS in the present pharmacological activity study. This model exhibits very similar clinical symptoms to human UC [109]. Firstly, bodyweight loss, DAI score, shortened colon length and spleen coefficient are frequently regarded as inflammatory signs to evaluate UC progression. It is also believed that colonic MPO activity is directly connected to the degree of neutrophil infiltration, which could cause the tissue damage at the site of UC inflammation. Our current investigation demonstrated that the intervention by SP may significantly reduce the above indexes in a dose-dependent way. Secondly, TNF-α triggers a wide range of inflammatory genes and encourages the production of pro-inflammatory cytokines [110]; IL-6 promotes neutrophil infiltration and results in tissue necrosis [111]; and iNOS produces excessive inflammatory mediators [112]. Namely, these mediators play a crucial role in the development of intestinal damage. Our findings also reinforced the significance of these inflammatory factors in the incidence and progression of ulcerative colitis, and also demonstrated that SP could drastically lower the iNOS, TNF-α and IL-6 levels in UC mice. Thirdly, oxidative stress is also involved in the pathogenesis of ulcerative colitis, with compelling evidence that the increased formation of reactive oxygen species damages cellular macromolecules and jeopardizes epithelial cell integrity. GSH, SOD and MDA are the most significant typical indicators for evaluating oxidative stress. To our satisfaction, SP treatment could dramatically reduce MDA concentrations, raise GSH levels and enhance SOD activity. Fourthly, the histopathology and transmission electron microscopy examination of colonic tissue are also important indexes to investigate the protective effect of SP on the intestinal barrier. As we expected, H&E staining and TEM revealed that SP could reduce the damage to the colonic intestinal barrier.

In order to further assess the effectiveness of SP and to investigate the relevant mechanisms, metabolomics analysis was carried out in this work. A total of 21 potential metabolite biomarkers and 11 metabolisms were identified to be closely related to the effect of SP. Network pharmacology analysis was then performed to screen out the active components (such as phenylpropanoids and flavonoids) and 373 potential biological targets. Aiming to establish the connection network between the biological targets and metabolites, integrated analysis, by merging metabolomics with network pharmacology, was finally employed. As a result, 10 metabolites out of 21 potential biomarkers were discovered to have a direct link with 18 biological targets among the 373 potential targets. Specifically, these ten metabolites were involved in five metabolisms. Three of these five pathways were lipid metabolism (arachidonic acid metabolism, linoleic acid metabolism and sphingolipid metabolism). Lipids influence the immune response by acting as intracellular and intercellular signaling molecules. It has been reported that lipid metabolism was expected to have a significant role in the pathophysiology of UC [113]. When colitis develops, the citrate cycle is disturbed, which reduces the amount of energy that the gut receives through aerobic breakdown. Tyrosine plays a critical role in the metabolism and development of both humans and animals and is linked to immunological activation and inflammation. To summarize, these 10 biomarkers, 18 targets and 5 metabolisms were thought to be critical in the therapeutic effect of SP on UC. It is believed that the substantial pharmacological effects of SP are due to its multi-target mechanism.

## 4. Materials and Methods

### 4.1. Materials and Reagents

The SP was collected in Shipeng Village, Panshi City, Jilin Province, China, in mid-September 2021. It was authenticated by Prof. Pingya Li as the whole herb of SP and was then air-dried. The specimen was preserved in the Natural Drugs Research Center of Jilin University.

The methanol and acetonitrile, of LC-MS grade, were bought from Fisher Chemical Company. The formic acid for UPLC was purchased from Sigma-Aldrich Company. All of the other chemicals were of analytical purity.

The phillygenin, pinoresinol diglucoside, luteolin 7-glucuronide, pinoresinol 4-glucoside, pinoresinol, isoquercitroside, 1,5-dicaffeoylquinic acid, matairesinoside, matairesinol and secoisolarieiresinol were purchased from ChemFaces. Chlorogenic acid, syringic acid, hispidulin, neochlorogenic acid, protocatechuic aldehyde, protocatechuic acid, 4,5-dicaffeoylquinic acid, 1,4-dicaffeoylquinic acid, eupafolin, quinic acid, cryptochlorogenic acid, rutin, caffeic acid, narcisin, quercitrin, arctiin, syringaresinol, apigenin, arctigenin, luteolin, dehydrocostus lactone, LPC (16:0), LPC (18:1), linolenic acid, linoleic acid and 9-oxo-10,12-octadecadienoic acid were purchased from Chengdu HerbSubstance Co., Ltd.

The DSS (MW: 40,000 Da) was purchased from Macklin Inc. Mouse MPO, the TNF-*α* and IL-6 ELISA kits were obtained from MultiSciences (Lianke) Biotech, Co., Ltd. The Mouse iNOS ELISA kit was purchased from Shanghai zcibio technology Co.,Ltd. The SOD, MDA, GSH assay kits were purchased from Nanjing Jiancheng Bioengineering Institute. Changyanning Tablet (Batch No. 2003044) was produced by Jiangxi Kang’enbei Traditional Chinese Medicine Co., Ltd.

### 4.2. Animals

Adult male BALB/c mice (22 ± 2 g) were bought from YISI Experimental Animal Technology Co., Ltd. (Changchun, China, License serial number: 202100040595). All of the mice were fed in the Observation Facility of Animal Experiment in Barrier Environment (SPF level, School of Basic Medicine, Jilin University) maintained under relative humidity (60 ± 5%) and standard temperature (25 ± 2 °C) with a 12 h light/dark cycle. After one week of acclimation, the mice were stochastically assigned to different experimental groups. In accordance with the Guide for Institutional Animal Care and Use of Laboratory Animals, the mice were kept in facilities approved by the Association for Institutional Animal Care and Use Committee of Jilin University.

### 4.3. Sample Preparation

*Ethanol extract of SP:* The dried whole herb of SP (1.0 kg) was extracted with 75% ethanol (10 L) for three times (3 h per time). The extracts were combined, and the ethanol was recovered by vacuum distillation, the obtained dried residue (ethanol extract of SP, 73.2 g) was stored at room temperature for further study.

*Test solution for qualitative analysis:* Ethanol extract was dissolved in methanol to obtain the solution at a concentration of 3.0 mg·mL^−1^.

*Test solutions for quantitative analysis:* (1) Ethanol extract was dissolved in methanol to obtain the solution at a concentration of 3.0 mg·mL^−1^; (2) Ethanol extract (70 mg) was suspended in water (30 mL), then extracted for three times with n-hexane (50 mL) and ethyl acetate (50 mL), respectively. The ethyl acetate layer was combined and recovered to dryness. The dried residue was then dissolved in methanol (1 mL) for test.

*Test solution for pharmacological activity test:* Ethanol extract was suspended in 0.5% sodium carboxymethylcellulose (CMC-Na) to prepare the solutions with the concentrations of 12.0, 6.0, 3.0 mg·mL^−1^.

### 4.4. UPLC-Q/TOF-MS

A Waters Acquity UPLC system connected to a Waters Xevo G2-XS QTOF mass spectrometer (Waters Co., Milford, MA, USA) was used to perform chromatographic separations and mass spectrometry detections via electrospray ionization interface. UPLC-MS/MS method was conducted as previously reported [114]. The details are shown in the Supporting Information.

### 4.5. Comprehensive Phytochemical Analysis

#### 4.5.1. Qualitative Analysis

Firstly, an independent database was created in addition to the Traditional Medicine Library within the UNIFI platform [30]. Namely, the chemical compositions reported for the *Saussurea* species were searched in online databases, including China National Knowledge Infrastructure (CNKI), Web of Science, ChemSpider, Medline and PubMed, and were gathered to form the database, including the names, chemical structures and molecular formulas of the components being acquired. Secondly, the MS raw data compressed by Waters Compression and Archival Tool v1.10, were imported into the UNIFI software (Waters, Manchester, UK) and were automatically analyzed by the workflow. The main parameters for the workflow were as follows: the minimum peak area was 200; the peak intensities of low and high energy were 200 and 1000 counts, respectively; the acceptable difference of retention time of reference substance was in the range of ±0.1 min. Both positive adducts (+H and +Na) and negative adducts (−H and +COOH) were selected in the analysis. The components that matched the evaluation criteria were screened quickly and were listed. Thirdly, the results were refined with a filter (mass error of the molecular weight or the typical fragments in the range of ±5 ppm, response value >5000). Finally, following the above conditions, the compound was identified by comparing the retention time and accurate molecular weight with the reference substance or by comparing the representative MS fragmentation patterns with the literature.

#### 4.5.2. Quantitative Analysis

The quantitative analysis of the SP ethanol extract was performed on polyphenols with representative skeletons, including 12 phenylpropanoids and 8 flavonoids, using UPLC-Q/TOF-MS. Three standard stock solutions (I~III) of mixtures were prepared in methanol: solution I contained chlorogenic acid, pinoresinol, luteolin, syringaresinol, 1,5-dicaffeoylquinic acid and pinoresinol 4-glucoside; solution II contained matairesinol, neochlorogenic acid, luteolin 7-glucuronide, isoquercitroside, 4,5-dicaffeoylquinic acid, matairesinoside and eupafolin; solution III contained rutin, 1,4-dicaffeoylquinic acid, narcisin, quercitrin, arctiin, apigenin and arctigenin.

Before the assay, a series of standard working solutions were created by properly diluting the stock solution. The external calibration method was used for the quantitative analysis. The validation of the method was as follows:

*Calibration curves* Each concentration of the mixed three standard solutions was injected and analyzed. The calibration curves were constructed by plotting the peak areas versus the concentrations.

*Limits of detection and quantification* The standard stocks were diluted with methanol to appropriate concentrations. The LOD and LOQ for each analyte were determined at S/N of about 3 and 10, respectively.

*Precision and accuracy* The method’s precision was assessed by intra- and inter-day variations. The standard solution was analyzed five times in a single day to calculate the intra-day precision, and the sample was analyzed multiple times over the course of six days to determine the inter-day precision. The recovery test was conducted to assess the method’s accuracy.

### 4.6. Alleviated Ulcerative Colitis Activity

#### 4.6.1. Experimental Design

In this study, Changyanning Tablet was used as a positive control drug [115]. After being fed adaptively for one week, the mice were randomly assigned to six groups (*n* = 10) consisting of control group, model group, Changyanning tablet group (CYN, 1.2 g·kg^−1^), low, middle and high dosages of SP ethanol extract groups (SPL, 30 mg·kg^−1^; SPM, 60 mg·kg^−1^; SPH, 120 mg·kg^−1^). From day 1 to day 7, the mice in control group were given normal water, while other five groups drank DSS aqueous solution (3.5%, *w/v*) *ad libitum* to induce UC model. From day 4 to day 10, the mice in the control and model groups were intragastrically administered with 0.5% sodium carboxymethylcellulose (CMC-Na) solution once a day, while the mice in the other groups were separately intragastrically administered with CYN or SP CMC-Na solution once a day. The volume of administration was all 10 mL/kg. All the mice were sacrificed on day 11 after fasting for 12 h, the blood and tissues were collected and explored for biochemical and histological changes.

#### 4.6.2. Body Weights, Clinical Signs Observations and Disease Activity Index (DAI)

On a daily basis, all mice were weighted and their general clinical signs including fecal characteristics and blood stool were recorded throughout the study period. DAI, obtained on the basis of the scores of weight loss, fecal characteristics and blood stool [116], was used to obtain a quantitative assessment.

#### 4.6.3. Sample Collection and Preparation

The blood obtained through eyeball enucleation was coagulated for half an hour and centrifuged (4000 rpm) for 15 min to obtain the serum samples for biochemical index determination. In addition, serum samples from control group, model group and SPH group were also used for metabolomic study.

After blood collection, the spleen and colon were flushed with PBS solution. The colon length (in terms of centimeters) and spleen coefficient (spleen weight (mg)/body weight (g)) were then calculated or measured for assessing the degree of inflammatory reaction. Then, the colons from each group were used to perform biochemical parameter determination, histological evaluation (fixed in 10% neutral-buffered formalin) and electron microscopy examinations (fixed in 2.5% glutaraldehyde). Moreover, the colons from the control, model and SPH groups were also used for the metabolomic study.

#### 4.6.4. Measurement of Cytokines and Myeloperoxidase (MPO) Contents

The homogenized colon samples were centrifuged for 10 min at 13,000 rpm at 4 °C after homogenization in PBS. TNF-α, iNOS and IL-6 levels in serum samples and in colon homogenate samples were measured using ELISA kits. In order to assess the activity of the neutrophils infiltrated into the colonic lamina, the MPO level in the colon homogenate sample was also evaluated using an ELISA kit.

#### 4.6.5. Measurement of Oxidative Stress Indexes Levels

According to the kit’s instructions, the activities of MDA, SOD and GSH in the colon homogenate samples were assessed.

#### 4.6.6. Histological Analysis

The colon tissue was sectioned, deparaffinized, hydrated and H&E stained after being fixed in 10% neutral-buffered formalin and paraffin-embedded. Photographs were taken of the colonic slides under a microscope.

#### 4.6.7. Transmission Electron Microscopy Examination

The fixed colon tissue was post-fixed in 1% OsO_4_, and then dehydrated through a graded ethanol series and embedded in epoxy resin. Uranyl acetate and lead citrate were used to counterstain ultrathin sections. Transmission electron microscopy (FEI Tecnai Spirit, USA) was used for observation and photography.

#### 4.6.8. Statistical Analysis

The SPSS 20.0 software was used for statistical analysis. The results were presented as Mean S.E.M. A one-way analysis of variance (ANOVA), followed by a Tukey test, was used to determine statistically significant difference (*p* < 0.05).

### 4.7. Metabolomics

Serum and colon samples of three groups of mice (control, model and SPH) were collected for metabolomic analysis (n = 10 mice in each group). The method for the metabolomic and data processing was conducted as previously reported [116]. The details are shown in the Supporting Information.

### 4.8. Network Pharmacology

The network pharmacology study was continued in order to explain the interactions between the phytochemicals and the pharmacological activity, and to predict the potential targets closely associated with the effect of SP from a comprehensive perspective. The method for network pharmacology was conducted as previously reported [117]. The details are shown in the Supporting Information.

### 4.9. Integrated Analysis Involving Metabolomics and Network Pharmacology

The potential biomarkers obtained from the metabolomics study and the potential targets obtained from the network pharmacology were used to perform the integrated analysis. Then, the “biomarkers-targets” correlation network was then constructed by using MetScape plugin (Cytoscape) based on the Metascape database (http://metascape.org/ (accessed on 11 October 2022)), DAVID database (https://david.ncifcrf.gov/ (accessed on 11 October 2022)) and Reactome database (https://reactome.org/ (accessed on 11 October 2022)). Finally, the intersection of the metabolisms from the integrated analysis and the metabolisms from the metabolomic study were screened out.

## 5. Conclusions

In the present study, the chemical composition and the pharmacological effect of SP 75% ethanol extract were investigated. A total of 149 components were qualitatively identified or tentatively identified from SP 75% ethanol extract. Among these, 139 components were identified from SP for the first time. Wherein, 12 phenylpropanoids and 8 flavonoids were quantitatively assayed and accounted for 33.2% of the ethanol extract of SP. The components with high contents were narcisin (6.94%), rutin (6.86%), arctiin (5.42%), chlorogenic acid (4.60%), apigenin (4.10%), 1,4-dicaffeoylquinic acid (2.04%) and pinoresinol (1.12%). Network pharmacology analysis showed that the phenylpropanoids and flavonoids contributed the most to the pharmacological activity of SP. By using the DSS-induced UC model mice, it was proven that SP 75% ethanol extract could dose-dependently alleviate bodyweight loss; decrease DAI score, spleen coefficient, levels of TNF-*α*, IL-6, iNOS, MPO and MDA; increase the colon length, GSH levels and SOD activity; and protect the intestinal barrier. A total of 10 biomarkers, 18 targets and 5 metabolisms were screened out to play vital roles in the therapeutic effect of SP on UC. To summarize, the SP 75% ethanol extract containing phenylpropanoids and flavonoids has a good anti-UC pharmacological effect, and it might be a viable candidate for alleviating UC symptoms.

## Figures and Tables

**Table 1:** Compounds identified from SP by UPLC-Q/TOF-MS.

NO.	*t_R_* (min)	Formula	Theoretical Mass (Da)	Calculated Mass (Da)	Mass Error (ppm)	MS^E^ Fragmentation	Identification	Ref.
1 *	0.71	C_7_H_12_O_6_	192.0634	192.0643	4.69	191.0570[M–H]^−^, 173.0443[M–H–H_2_O]^−^	Quinic acid	S
2 *	0.79	C_16_H_18_O_9_	354.0951	354.0961	2.82	353.0988[M–H]^−^, 191.0569[M–H–C_9_H_6_O_3_]^−^, 179.0338[M–H–C_7_H_10_O_5_]^−^, 135.0451[M–H–C_8_H_10_O_7_]^−^	Chlorogenic acid	S
3 *	0.82	C_6_H_12_O_6_	180.0634	180.0637	1.67	179.0564[M–H]^−^, 131.0358[M–H–H_2_O–CH_2_O]^−^, 103.0404[M–H–H_2_O–C_2_H_5_O_2_]^−^	D-Galactose	[21]
4 *	0.83	C_30_H_38_O_15_	638.2211	638.2232	3.29	683.2214[M–H]^−^, 489.1377[M–H–Fuc]^−^, 458.1828[M–H–C_9_H_7_O_4_]^−^, 161.0201[M–H–Glu–Fuc–C_8_H_11_O_2_]^−^	Sucrose	[22]
5 *	0.83	C_12_H_22_O_11_	342.1162	342.1179	4.97	341.1106[M–H]^−^, 179.0571[M–H–Glu]^−^, 161.0465[M–H–OFru]^−^	Cistanoside C	[23]
6 *	0.94	C_10_H_17_NO_3_	199.1208	199.1213	2.51	200.1286[M+H]^+^, 168.1017[M+H–CH_3_O]^+^, 126.0930[M+H–C_2_H_4_–H_2_O–CH_3_O]^+^, 122.0978[M+H–H_2_O–C_2_H_3_O_2_]^+^, 94.0687[M+H–C_2_H_4_–H_2_O–C_2_H_3_O_2_]^+^	Tussilagine	[24]
7 *	0.96	C_14_H_18_O_9_	330.0951	330.0961	3.03	329.0889[M–H]^−^, 167.0356[M–H–Glu]^−^	Mudanoside A	[25]
8 *	0.98	C_13_H_16_O_10_	332.0743	332.0754	3.31	331.0681[M–H]^−^, 168.0068[M–H–Glu]^−^, 124.0173[M–H–Glu–CO_2_]^−^	Glucogallin	[26]
9 *	1.01	C_7_H_6_O_4_	154.0266	154.0264	–1.30	153.0192[M–H]^−^, 109.0281[M–H–HCOOH]^−^	Protocatechuic acid	S
10 *	1.03	C_11_H_12_O_6_	240.0634	240.0643	3.75	285.0625[M+HCOO]^−^, 239.0564[M–H]^−^, 149.0597[M–H–2HCOOH]^−^, 108.0518[M–H–C_4_H_2_O_5_]^−^	Eucomic acid	[27]
11 *	1.08	C_15_H_21_NO_7_	327.1318	327.1323	1.53	328.1395[M+H]^+^, 310.1288[M+H–H_2_O]^+^, 292.1183[M+H–2H_2_O]^+^, 264.1229[M+H–H_2_O–HCOO]^+^, 166.0867[M+H–C_6_H_10_O_5_]^+^	Fructose-phenylalanine	[28]
12 *	1.13	C_14_H_20_O_9_	332.1107	332.1121	4.22	331.1049[M–H]^−^, 168.0431[M–H–Glu]^−^, 154.0237[M–H–Glu–CH_3_]^−^, 139.0028[M–H–Glu–2CH_3_]^−^, 137.0246[M–H–Glu–CH_3_O]^−^	Leonuriside A	[29]
13 *	1.16	C_16_H_18_O_9_	354.0951	354.0964	3.75	353.0887[M–H]^−^, 191.0568[M–H–C_9_H_6_O_3_]^−^, 135.0456[M–H–C_8_H_10_O_7_]^−^	Neochlorogenic acid	S
14 *	1.20	C_15_H_18_O_9_	342.0951	342.0965	4.09	341.0892[M–H]^−^, 179.0342[M–H–Glu]^−^, 135.0446[M–H–Glu–CO_2_]^−^	Phoeniceoside	[30]
15 *	1.24	C_15_H_18_O_8_	326.1002	326.1011	2.76	325.0938[M–H]^−^, 163.0413[M–H–Glu]^−^, 119.0513[M–H–Glu–HCOOH]^−^	Melilotoside	[31]
16 *	1.30	C_18_H_18_O_5_	314.1154	314.1162	3.18	315.1235[M+H]^+^, 193.0875[M+H–C_7_H_6_O_2_]^+^, 147.0451[M+H–CH_3_O–C_8_H_9_O_2_]^+^, 137.0622[M+H–C_10_H_10_O_3_]^+^	*p*-Hydroxyphenethyl ferulate	CFM-ID
17 *	1.31	C_7_H_6_O_4_	154.0266	154.0270	2.60	153.0197[M–H]^−^, 109.0293[M–H–HCOOH]^−^	3,4-Dihydroxybenzoic acid	[32]
18 *	1.45	C_9_H_7_NO	145.0528	145.0523	−3.45	146.0595[M+H]^+^, 118.0655[M+H–CHO]^+^	Indole-3-aldehyde	[33]
19 *	1.47	C_27_H_28_N_2_O_4_	444.2049	444.2035	−3.16	443.1962[M–H]^−^, 252.1025[M–H–C_11_H_13_O_2_N]^−^	Cryptochlorogenic acid	S
20 *	1.47	C_16_H_18_O_9_	354.0951	354.0962	3.11	353.0889[M–H]^−^, 307.0824[M–H–HCOOH]^−^, 191.0566[M–H–C_9_H_6_O_3_]^−^, 146.0587[M–H–C_9_H_6_O_3_–HCOOH]^−^	Aurantiamide acetate	[34]
21 *	1.67	C_10_H_12_O_4_	196.0736	196.0742	3.06	241.0724[M+HCOO]^−^, 195.0661[M–H]^−^, 179.0721[M–H–H_2_O]^−^, 165.0563[M–H–CH_3_O]^−^	Acetosyringone	[35]
22 *	1.76	C_18_H_26_O_9_	386.1577	386.1590	3.37	431.1572[M+HCOO]^−^, 385.1503[M–H]^−^, 223.0995[M–H–Glu]^−^, 135.0467[M–H–OGlu–C_4_H_7_O]^−^	Methylsyringin	[36]
23	1.78	C_19_H_27_NO_6_	365.1838	365.1844	1.62	366.1917[M+H]^+^, 330.1704[M+H–2H_2_O]^+^, 262.1437[M+H–H_2_O–C_4_H_7_O_2_]^+^	Pulchellamine B	[37]
24 *	1.82	C_14_H_18_O_7_	298.1053	298.1067	4.70	343.1039[M+HCOO]^−^, 164.0695[M–H–C_8_H_7_O_2_]^−^, 133.0303[M–H–Glu]^−^, 121.0300[M–H–Glu–CH_3_]^−^	Ameliaroside	[38]
25 *	1.88	C_20_H_27_NO_6_	377.1838	377.1846	2.12	378.1919[M+H]^+^, 360.1802[M+H–H_2_O]^+^, 332.1862[M+H–HCOOH]^+^, 314.1749[M+H–HCOOH–H_2_O]^+^, 227.1060[M+H–2H_2_O–C_5_H_9_NO_2_]^+^	Calophyllamine A	[39]
26	1.97	C_17_H_24_O_9_	372.1420	372.1433	3.49	417.1445[M+HCOO]^−^, 371.1351[M–H]^−^, 209.0821[M–H–Glu]^−^, 194.0586[M–H–CH_3_–Glu]^−^, 151.0409[M–H–Glu–C_3_H_5_O]^−^	Syringin	[26]
27 *	2.08	C_20_H_20_O_8_	388.1158	388.1156	−0.51	411.1048 [M+Na]^+^, 389.1232[M+H]^+^, 371.1133[M+H–H_2_O]^+^, 167.0720[M+H–C_11_H_10_O_5_]^+^	6*α*-Catechyl-2*α*-guaicyl-3,7-dioxabicyclo [3.3.0]octan-4-one	[40]
28 *	2.26	C_7_H_6_O_3_	138.0317	138.0323	4.35	137.0251[M–H]^−^, 109.0302[M–H–CHO]^−^	Protocatechuic aldehyde	S
29 *	2.36	C_15_H_20_O_8_	328.1158	328.1168	3.05	327.1095[M–H]^−^, 165.0562[M–H–Glu]^−^, 147.0453[M–H–H_2_O–Glu]^−^	Paeonoside	[41]
30 *	2.48	C_17_H_24_O_10_	388.1369	388.1387	4.61	387.1305[M–H]^−^, 371.0989[M–H–CH_3_]^−^, 207.0664[M–H–OGlu]^−^, 192.0432[M–H–Glu–CH_3_O]^−^	Geniposide	[42]
31 *	2.57	C_16_H_18_O_8_	338.1002	338.1010	2.37	337.0931[M–H]^−^, 191.0562[M–H–C_9_H_7_O_2_]^−^, 163.0402[M–H–C_7_H_11_O_5_]^−^	3-*p*-Coumaroylquinic acid	[43]
32 *	2.61	C_8_H_8_O_4_	168.0423	168.0426	1.79	167.0373[M–H]^−^, 123.0355[M–H–HCOOH]^−^, 108.0216[M–H–HCOOH–CH_3_]^−^, 93.0343[M–H–HCOOH–CH_3_O]^−^	Vanillic acid	[44]
33 *	2.77	C_9_H_10_O_5_	198.0528	198.0551	1.51	197.0449[M–H]^−^, 179.0345[M–H–H_2_O]^−^, 135.0444[M–H–H_2_O–HCOOH]^−^	Syringic acid	[45]
34 *	2.80	C_17_H_26_O_7_	342.1679	342.1692	3.80	387.1664[M+HCOO]^−^, 341.1608[M–H]^−^, 163.1127[M–H–OGlu]^−^	Jasmolone glucoside	CFM-ID
35 *	2.81	C_9_H_8_O_4_	180.0423	180.0422	−0.56	179.0340[M–H]^−^, 135.0438[M–H–HCOOH]^−^	Caffeic acid	S
36 *	2.82	C_20_H_27_NO_6_	377.1838	377.1831	−1.92	378.1904[M+H]^+^, 332.1854[M+H–HCOOH]^+^, 257.1408[M+H–2H_2_O–CH_2_–C_3_H_3_O_2_]^+^, 235.0971[M+H–C_3_H_3_O_2_–C_4_H_8_N]^+^, 206.0939[M+H–C_3_H_3_O_2_–C_5_H_8_NO]^+^	Lanicepomine A	[37]
37 *	2.98	C_13_H_18_O_6_	270.1103	270.1111	2.96	315.1113[M+HCOO]^−^, 269.1029[M–H]^−^, 161.0455[M–H–C_7_H_8_O]^−^	Benzyl *β*-D-glucoside	[46]
38 *	3.06	C_15_H_16_O_6_	292.0947	292.0958	3.77	337.0930[M+HCOO]^−^, 291.0873[M–H]^−^, 163.0414[M–H–H_2_O–C_2_HO–C_4_H_5_O]^−^	Cnidimol D	[47]
39 *	3.50	C_11_H_14_O_5_	226.0841	226.0844	1.34	225.0770[M–H]^−^, 195.0663[M–H–CH_3_O]^−^, 180.0427[M–H–C_2_H_5_O]^−^, 149.0240[M–H–CH_3_O–C_2_H_5_O]^−^	3-Hydroxy-1-(4-hydroxy-3,5-dimethoxyphenyl)propan-1-one	[48]
40 *	3.52	C_26_H_34_O_12_	538.2050	538.2058	1.49	583.2031[M+HCOO]^−^, 537.1982[M–H]^−^, 375.1454[M–H–Glu]^−^, 357.1342[M–H–Glu–H_2_O]^−^, 151.0407[M–H–Glu–C_12_H_16_O_4_]^−^	Medusaside A	[49]
41 *	3.58	C_17_H_20_O_9_	368.1107	368.1110	0.81	367.1037[M–H]^−^, 191.0564[M–H–CH_3_–C_9_H_5_O_3_]^−^, 161.0241[M–H–C_8_H_14_O_6_]^−^, 135.0450[M–H–C_9_H_12_O_7_]^−^	Methyl 3-caffeoylquinate	[50]
42 *	3.70	C_19_H_32_O_8_	388.2097	388.2116	4.89	433.2099[M+HCOO]^−^, 387.2030[M–H]^−^, 225.1501[M–H–Glu]^−^, 153.0920[M–H–C_4_H_7_O–Glu]^−^	Icariside B8	CFM-ID
43 *	3.72	C_26_H_34_O_12_	538.2050	538.2068	3.34	583.2039[M+HCOO]^−^, 537.1980[M–H]^−^, 375.1451[M–H–Glu]^−^, 153.0927[M–H–C_17_H_20_O_10_]^−^	Medusaside B	[49]
44 *	3.73	C_19_H_30_O_8_	386.1941	386.1949	2.07	431.1961 [M+HCOO]^−^, 385.1970[M–H]^−^, 223.1344[M–H–Glu]^−^, 205.1231[M–H–Glu–H_2_O]^−^	Saussureoside B	[51]
45	4.21	C_20_H_29_NO_6_	379.1995	379.2002	1.85	380.2075[M+H]^+^, 334.2013[M+H–HCOOH]^+^, 316.1910[M+H–HCOOH–H_2_O]^+^, 215.1075[M+H–2H_2_O–C_6_H_11_NO_2_]^+^	Pulchellamine E	[37]
46 *	4.29	C_9_H_10_O_3_	166.0630	166.0635	3.01	165.0562[M–H]^−^, 147.0452[M–H–H_2_O]^−^	Phloretic acid	[52]
47 *	4.37	C_26_H_34_O_12_	538.2050	538.2069	3.53	537.1986[M–H]^−^, 375.1451[M–H–Glu]^−^, 327.1240[M–H–Glu–H_2_O–CH_3_O]^−^, 297.1136[M–H–Glu–H_2_O-2CH_3_O]^−^, 225.1250[M–H–Glu–C_8_H_7_O_3_]–	Lanicepside A	[53]
48 *	4.44	C_8_H_8_O_2_	136.0524	136.0527	2.21	135.0455[M–H]^−^, 120.0213[M–H–CH_3_]^−^, 92.0267[M–H–C_2_H_3_O]^−^	Curculigoside C	[54]
49 *	4.44	C_22_H_26_O_12_	482.1424	482.1439	3.11	481.1346[M–H]^−^, 197.0455[M–H–Glu– C_7_H_5_O_2_]^−^, 121.0295[M–H–Glu–C_9_H_9_O_5_]^−^	*p*-Hydroxyacetophenone	[55]
50 *	4.49	C_21_H_34_O_9_	430.2203	430.2220	3.95	429.2127[M–H]^−^,401.1817[M–H–C_2_H_4_]^−^, 267.1603[M–H–Glu]^−^	4*α*(15),11*β*(13)-Tetrahydroridentin B-1 -glucoside	[56]
51 *	4.56	C_26_H_34_O_12_	538.2050	538.2067	3.16	537.2064[M–H]^−^, 327.1240[M–H–Glu–H_2_O–CH_3_O]^−^, 195.0664[M–H–Glu–C_10_H_11_O_3_]^−^, 161.0464[M–H–C_20_H_24_O_7_]^−^	Citrusin A	[57]
52 *	4.64	C_26_H_34_O_12_	538.2050	538.2070	3.72	583.2048[M+HCOO]^−^, 537.1982[M–H]^−^, 375.1442[M–H–Glu]^−^, 327.1245[M–H–Glu–H_2_O–CH_3_O]^−^, 179.0561[M–H–C_20_H_22_O_6_]^−^	Lanicepside B	[53]
53 *	4.66	C_32_H_42_O_16_	682.2473	682.2499	3.81	727.2481[M+HCOO]^−^, 681.2411[M–H]^−^, 519.1877[M–H–Glu]^−^, 339.1242[M–H–2Glu–H_2_O]^−^	Pinoresinol diglucoside	S
54 *	4.73	C_27_H_36_O_13_	568.2156	568.2166	1.76	613.2188[M+HCOO]^−^, 567.2092[M–H]^−^, 521.2040[M–H–H_2_O–CH_3_O]^−^, 405.1565[M–H–Glu]^−^, 195.0662[M–H–Glu–C_11_H_13_O_4_]^−^	Citrusin B	CFM-ID
55 *	5.01	C_26_H_36_O_11_	524.2258	524.2240	−3.43	523.2167[M–H]^−^, 507.1880[M–H–CH_3_]^−^, 361.1690[M–H–Glu]^−^, 346.1771[M–H–Glu–CH_3_]^−^, 315.1331[M–H–Glu–CH_3_–CH_3_O]^−^	(-)-Secoisolariciresinol-4-O-*β*-D-glucoside	[58]
56 *	5.01	C_17_H_20_O_9_	368.1107	368.1116	2.44	367.1033[M–H]^−^, 179.0346[M–H–C_8_H_12_O_5_]^−^, 161.0247[M–H–C_8_H_14_O_6_]^−^, 135.0472[M–H–C_9_H_12_O_7_]^−^	Methyl 4-caffeoylquinate	[59]
57 *	5.08	C_19_H_24_O_8_	380.1471	380.1484	3.42	425.1466[M+HCOO]^−^, 379.1404[M–H]^−^, 343.1188[M–H–2H_2_O]^−^	15-Hydroxyjanerin	CFM-ID
58	5.11	C_21_H_31_NO_6_	393.2151	393.2163	3.05	416.2095[M+Na]^+^, 394.2241[M+H]^+^, 378.1929[M+H–CH_3_]^+^, 342.1711[M+H–CH_3_–2H_2_O]^+^, 262.1448[M+H–C_6_H_12_O_2_–H_2_O]^+^, 228.1161[M+H–C_6_H_12_NO_2_–2H_2_O]^+^	Pulchellamine G	[37]
59 *	5.21	C_28_H_38_O_13_	582.2312	582.2303	−1.55	581.2230[M–H]^−^, 419.1720[M–H–Glu]^−^, 389.1603[M–H–Glu–CH_3_O]^−^, 373.1298[M–H–Glu–CH_3_–CH_3_O]^−^	Lyoniresinol-3*α*-glucoside	[60]
60 *	5.28	C_21_H_18_O_12_	462.0798	462.0806	1.73	461.0734[M–H]^−^, 285.0404[M–H–Gluac]^−^, 151.0049[M–H–Gluac–C_8_H_6_O_2_]^−^, 132.0210[M–H–Gluac–C_7_H_4_O_4_]^−^	Luteolin 7-glucuronide	S
61	5.32	C_27_H_30_O_16_	610.1534	610.1530	−0.66	609.1457[M–H]^−^, 461.0731[M–H–Rha]^−^, 300.0281[M–H–Glu–Rha]^−^	Rutin	S
62 *	5.57	C_21_H_20_O_12_	464.0955	464.0978	4.96	463.0906[M–H]^−^, 300.0280[M–H–Glu]^−^, 151.0041[M–H–Glu–C_8_H_5_O_3_]^−^, 150.0328[M–H–Glu–C_7_H_4_O_4_]^−^	Isoquercitroside	S
63 *	5.62	C_19_H_22_O_5_	330.1467	330.1475	2.42	375.1447[M+HCOO]^−^, 329.1395[M–H]^−^, 297.1131[M–H–CH_3_–H_2_O]^−^, 282.0899[M–H–CH_3_–CH_2_–H_2_O]^−^, 226.0641[M–H–H_2_O–CH_2_–C_4_H_7_O]^−^	Aguerin B	[61]
64 *	5.66	C_27_H_34_O_12_	550.2050	550.2069	3.45	595.2042[M+HCOO]^−^, 549.1984[M–H]^−^, 519.1876[M–H–CH_3_O]^−^, 387.1454[M–H–Glu]^−^	Saussurenoside	[62]
65 *	5.72	C_15_H_10_O_7_	302.0427	302.0435	2.65	303.0508[M+H]^+^, 178.0272[M+H– C_6_H_5_O_3_]^+^, 153.0195[M+H–C_8_H_5_O_3_]^+^, 108.0216[M+H–H2O–C_9_H_5_O_4_]^+^	Isoetin	[63]
66 *	5.76	C_25_H_24_O_12_	516.1268	516.1287	3.68	515.1204[M–H]^−^, 353.0885[M–H–C_9_H_6_O_3_]^−^, 335.0776[M–H–C_9_H_9_O_4_]^−^, 191.0570[M–H–2C_9_H_6_O_3_]^−^, 179.0353[M–H–C_16_H_16_O_8_]^−^	1,4-Dicaffeoylquinic acid	S
67 *	5.96	C_27_H_30_O_15_	594.1585	594.1598	2.19	593.1515[M–H]^−^, 285.0407[M–H–Rut]^−^	Luteolin-7-rutinoside	[64]
68 *	6.03	C_25_H_24_O_12_	516.1268	516.1271	0.58	515.1198[M–H]^−^, 353.0878[M–H–C_9_H_6_O_3_]^−^, 191.0561[M–H–2C_9_H_6_O_3_]^−^, 179.0352[M–H–C_16_H_16_O_8_]^−^	1,5-Dicaffeoylquinic acid	S
69 *	6.06	C_28_H_32_O_16_	624.1690	624.1720	4.81	623.1647[M–H]^−^, 351.0735[M–H–ORha–C_6_H_4_O_2_]^−^, 315.0530[M–H–Rut]^−^	Narcisin	S
70 *	6.15	C_27_H_30_O_14_	578.1636	578.1653	2.94	577.1580[M–H]^−^, 269.0474[M–H–Neo]^−^	Rhoifolin	[65]
71 *	6.18	C_21_H_18_O_11_	446.0849	446.0868	4.26	445.0791[M–H]^−^, 284.0322[M–H–Glu]^−^, 269.0464[M–H– OGlu]^−^	Rhein-8-glucoside	[66]
72	6.29	C_22_H_26_O_8_	418.1628	418.1639	2.63	417.1567[M–H]^−^, 402.1271[M–H–CH_3_]^−^, 387.1080[M–H–2CH_3_]^−^, 181.0521[M–H–C_13_H_11_O_4_]^−^	Syringaresinol	S
73	6.29	C_21_H_20_O_11_	448.1006	448.1021	3.35	447.0938[M–H]^−^, 301.0375[M–H–Rha]^−^, 283.0255[M–H–Rha–H_2_O]^−^, 151.0043[M–H–Rha–C_8_H_5_O_3_]^−^	Quercitrin	S
74	6.31	C_26_H_32_O_12_	536.1894	536.1901	1.31	535.1823[M–H]^−^, 501.1768[M–H–2H_2_O]^−^, 355.1188[M–H–Glu–H_2_O]^−^, 151.0405[M–H–Glu–C_12_H_13_O_4_]^−^	1-Hydroxypinoresinol-1 -glucoside	[67]
75 *	6.36	C_21_H_20_O_10_	432.1056	432.1075	4.40	477.1057[M+HCOO]^−^, 431.0993[M–H]^−^, 285.0405[M–H–Rha]^−^, 161.0464[M–H–C_15_H_10_O_5_]^−^	Afzelin	[68]
76 *	6.45	C_25_H_24_O_12_	516.1268	516.1292	4.65	515.1219[M–H]^−^, 353.0891[M–H–C_9_H_6_O_3_]^−^, 191.0579[M–H–2C_9_H_6_O_3_]^−^, 179.0359[M–H–C_16_H_16_O_8_]^−^	4,5-Dicaffeoylquinic acid	S
77 *	6.47	C_21_H_20_O_10_	432.1056	432.1071	3.47	431.0988[M–H]^−^, 269.0461[M–H–Glu]^−^	Cosmosiin	[68]
78 *	6.57	C_26_H_32_O_11_	520.1945	520.1931	−2.71	565.1913[M+HCOO]^−^, 519.1851[M–H]^−^, 357.1323[M–H–Glu]^−^, 151.0387[M–H–Glu–C_12_H_13_O_3_]^−^	Pinoresinol 4- glucoside	S
79 *	6.63	C_9_H_16_O_4_	188.1049	188.1050	0.53	187.0977[M–H]^−^, 143.1081[M–H–HCOOH]^−^, 125.0968[M–H–H_2_O–HCOOH]^−^	Azelaic acid	[69]
80 *	6.67	C_22_H_22_O_11_	462.1162	462.1151	−2.38	461.1078[M–H]^−^, 446.0853[M–H–CH_3_]^−^, 298.0472[M–H–Glu]^−^, 283.0244[M–H–Glu–CH_3_]^−^	Thermopsoside	[70]
81 *	6.82	C_34_H_30_O_15_	678.1585	678.1606	3.10	677.1513[M–H]^−^, 515.1194[M–H–C_9_H_6_O_3_]^−^, 497.1098[M–H–C_9_H_8_O_4_]^−^, 353.0881[M–H–2C_9_H_6_O_3_]^−^, 179.0346[M–H–C_25_H_22_O_11_]^−^	1,3,5-Tricaffeoylquinic acid	[71]
82 *	6.89	C_20_H_26_O_8_	394.1628	394.1641	3.30	417.1533[M+Na]^+^, 395.1713[M+H]^+^, 359.1508[M+H–2H_2_O]^+^, 350.1378[M+H–C_2_H_5_O]^+^, 327.1243[M+H–2H_2_O–CH_3_O]^+^, 229.0776[M+H–C_2_H_5_O–H_2_O–C_4_H_7_O_3_]^+^	Methoxyjanerin	[72]
83 *	6.93	C_20_H_26_O_6_	362.1729	362.1737	2.21	361.1664[M–H]^−^, 346.1428[M–H–CH_3_]^−^, 327.1231[M–H–H_2_O–CH_3_]^−^, 315.1247[M–H–CH_3_–CH_3_O]^−^, 165.0563[M–H–C_10_H_13_O_3_–CH_3_]^−^	Secoisolariciresinol	S
84 *	7.00	C_21_H_22_O_7_	386.1366	386.1361	−1.29	387.1434[M+H]^+^, 163.0400[M+H–C_10_H_10_O_4_–2CH_3_]^+^, 135.0453[M+H–C_13_H_16_O_5_]^+^	Conicaol B	[73]
85 *	7.06	C_26_H_32_O_11_	520.1945	520.1959	2.69	519.1876[M–H]^−^, 357.1345[M–H–Glu]^−^, 342.1116[M–H–Glu–CH_3_]^−^, 121.0305[M–H–Glu–C_13_H_15_O_4_]^−^	Matairesinoside	S
86 *	7.09	C_20_H_28_O_7_	380.1835	380.1852	4.47	425.1827[M+HCOO]^−^, 379.1772[M–H]^−^, 221.0840[M–H–C_8_H_14_O_3_]^−^, 209.0834[M–H–C_9_H_14_O_3_]^−^	Elemacarmanin	CFM-ID
87 *	7.10	C_18_H_22_O_6_	334.1416	334.1421	1.50	357.1353[M+Na]^+^, 335.1502[M+H]^+^, 317.1404[M+H–H_2_O]^+^, 137.0614[M+H–H_2_O–C_8_H_9_O–C_2_H_3_O_2_]^+^	7α-Hydroxygerin	[74]
88 *	7.14	C_20_H_22_O_4_	326.1518	326.1524	1.84	327.1597[M+H]^+^, 203.1089[M+H–C_7_H_8_O_2_]^+^, 189.0924[M+H–C_7_H_7_O_2_–CH_3_]^+^, 137.0614[M+H–C_12_H_14_O_2_]^+^	Dehydrodiisoeugenol	[75]
89 *	7.28	C_22_H_24_O_8_	416.1471	416.1469	−0.48	417.1542[M+H]^+^, 399.1435[M+H–H_2_O]^+^, 358.1362[M+H–C_2_H_2_O_2_]^+^, 137.0613[M+H–H_2_O–C_14_H_14_O_5_]^+^	Acetoxypinoresinol	CFM-ID
90 *	7.35	C_30_H_34_O_10_	554.2152	554.2171	3.43	553.2089[M–H]^−^, 535.1990[M–H–H_2_O]^−^, 357.1352[M–H–H_2_O–C_10_H_10_O_3_]^−^, 181.0877[M–H–C_20_H_20_O_7_]^−^	Lappaol E	[76]
91 *	7.49	C_18_H_18_O_3_	282.1256	282.1267	3.90	327.1249[M+HCOO]^−^, 239.0726[M–H–C_3_H_6_]^−^, 197.0626[M–H–C_3_H_5_–C_2_H_3_–H_2_O]^−^, 163.0405[M–H–C_9_H_10_]^−^	Obovatol	[77]
92 *	7.50	C_20_H_20_O_5_	340.1311	340.1321	2.94	339.1248[M–H]^−^, 324.1008[M–H–CH_3_]^−^, 293.0825[M–H–CH_3_–CH_3_O]^−^, 265.0519[M–H–H_2_O–C_4_H_8_]^−^	Licocoumarone	[78]
93 *	7.51	C_20_H_22_O_6_	358.1416	358.1425	2.51	357.1342[M–H]^−^, 342.1117[M–H–CH_3_]^−^, 151.0405[M–H–C_12_H_16_O_3_]^−^, 136.0538[M–H–C_12_H_11_O_3_–H_2_O]^−^	Pinoresinol	S
94 *	7.68	C_30_H_34_O_10_	554.2152	554.2168	2.89	553.2095[M–H]^−^,535.1954[M–H–H_2_O]^−^, 517.1888[M–H–2H_2_O]^−^	Lappaol C	[79]
95 *	7.72	C_17_H_20_O_4_	288.1362	288.1376	4.86	311.1268[M+Na]^+^, 289.1457[M+H]+, 230.1312[M+H–C_2_H_3_O_2_]^+^, 202.1370[M+H–C_2_H_3_O_2_–CO]^+^	8*α*-Acetoxydehydrocostuslactone	[80]
96 *	8.00	C_27_H_34_O_11_	534.2101	534.2118	3.18	579.2090[M+HCOO]^−^, 533.2035[M–H]^−^, 371.1512[M–H–Glu]^−^, 356.1280[M–H–Glu–CH_3_]^−^, 136.0535[M–H–Glu–C_13_H_14_O_4_]^−^, 121.0306[M–H–Glu–CH_3_–C_13_H_14_O_4_]^−^	Arctiin	S
97 *	8.18	C_15_H_10_O_6_	286.0477	286.0488	3.85	285.0415[M–H]^−^, 151.0044[M–H–C_8_H_6_O_2_]^−^, 133.0308[M–H–C_7_H_4_O_4_]^−^, 107.0144[M–H–C_9_H_6_O_4_]^−^	Luteolin	S
98 *	8.23	C_8_H_8_O_2_	136.0524	136.0521	−2.21	137.0613[M+H]^+^, 122.0364[M+H–CH_3_]^+^, 94.0407[M+H–C_2_H_3_O]^+^	Phenyl acetate	[81]
99 *	8.24	C_34_H_30_O_15_	678.1585	678.1614	4.28	677.1521[M–H]^−^, 515.1210[M–H–C_9_H_6_O_3_]^−^, 353.0895[M–H–2C_9_H_6_O_3_]^−^, 335.0788[M–H–C_9_H_7_O_3_–C_9_H_7_O_4_]^−^, 179.0352[M–H–C_25_H_22_O_11_]^−^	3,4,5–Tricaffeoylquinic acid	[82]
100 *	8.26	C_21_H_24_O_6_	372.1573	372.1583	2.69	373.1656[M+H]^+^, 355.1549[M+H–H_2_O]^+^, 137.0617[M+H–C_13_H_16_O_4_]^+^, 122.0386[M+H–C_14_H_19_O_4_]^+^	Phillygenin	S
101 *	8.46	C_30_H_36_O_9_	540.2359	540.2381	4.07	585.2352[M+HCOO]^−^, 539.2308[M–H]^−^, 521.2194[M–H–H_2_O]^−^, 509.2192[M–H–CH_3_O]^−^, 371.1505[M–H–CH_3_O–C_8_H_9_O_2_]^−^, 297.1145[M–H–H_2_O–C_12_H_16_O_4_]^−^	Sesquipinsapol B	[83]
102 *	8.54	C_16_H_12_O_7_	316.0583	316.0596	4.11	317.0668[M+H]^+^, 302.0429 [M+H–CH_3_]^+^, 168.0062[M+H–CH_3_–C_8_H_6_O_2_]^+^, 140.0506[M+H–C_9_H_5_O_4_]^+^	Eupafolin	S
103 *	9.04	C_15_H_24_O_2_	236.1776	236.1786	4.23	237.1858[M+H]^+^, 219.1771[M+H–H_2_O]^+^, 108.0945[M+H–C_7_H_13_O_2_]^+^, 92.0631[M+H–C_3_H_7_O–H_2_O–C_5_H_8_]^+^	Eudesma-4(14),11(13)-diene-3*β*,12-diol	[84]
104 *	9.08	C_31_H_36_O_10_	568.2308	568.2328	3.52	567.2256[M–H]^−^, 535.1982[M–H–CH_3_O]^−^, 517.1888[M–H–H_2_O–CH_3_O]^−^, 191.0714[M–H–C_20_H_24_O_7_]^−^	Lappaol D	[79]
105 *	9.28	C_34_H_37_N_3_O_6_	583.2682	583.2684	0.34	584.2757[M+H]^+^, 438.2385[M+H–C_9_H_6_O_2_]^+^, 292.2026[M+H–2C_9_H_6_O_2_]^+^, 275.1765[M+H–C_9_H_6_O2–C_9_H_9_NO_2_]^+^, 147.0453[M+H–C_25_H_31_N_3_O_4_]^+^	*N*1,*N*5,*N*10-Tri-*p*-coumaroylspermidine	[85]
106 *	9.33	C_15_H_10_O_5_	270.0528	270.0539	4.07	269.0456[M–H]^−^, 151.0039[M–H–C_8_H_6_O]^−^, 117.0356[M–H–C_7_H_8_O_4_]^−^, 107.0145[M–H–C_9_H_6_O_3_]^−^	Apigenin	S
107	9.38	C_26_H_30_N_2_O_6_	466.2104	466.2087	−3.65	489.1989[M+Na]^+^, 467.2160[M+H]^+^, 321.1205[M+H–CH_3_–C_9_H_9_N]^+^, 303.1119[M+H–H_2_O–CH_3_–C_9_H_9_N]^+^, 265.1430[M+H–C_11_H_10_N_2_O_2_]^+^, 202.0747[M+H–C_15_H_21_O_4_]^+^	Pulchellamine F	[37]
108 *	9.47	C_20_H_22_O_6_	358.1416	358.1431	4.19	357.1348[M–H]^−^, 342.1113[M–H–CH_3_]^−^, 179.0718[M–H–C_10_H_10_O_3_]^−^, 165.0563[M–H–C_10_H_9_O_3_–CH_3_]^−^, 122.0370[M–H–C_13_H_15_O_4_]^−^	Matairesinol	S
109 *	9.61	C_16_H_12_O_6_	300.0634	300.0640	2.00	299.0568[M–H]^−^, 284.0330[M–H–CH_3_]^−^, 256.0384[M–H–C_2_H_3_O]^−^, 161.0246[M–H–C_7_H_6_O_3_]^−^	Hispidulin	S
110 *	9.71	C_18_H_22_O_5_	318.1467	318.1476	2.83	341.1378[M+Na]^+^, 319.1556[M+H]^+^, 287.1297[M+H–CH_3_O]^+^, 189.0917[M+H–C_2_H_4_O–C_4_H_6_O_2_]^+^	Gerin	[74]
111 *	9.81	C_18_H_32_O_5_	328.2250	328.2260	3.05	327.2228[M–H]^−^, 291.1969[M–H–2H_2_O]^−^, 229.1455[M–H–C_6_H_10_O]^−^, 183.1392[M–H–H_2_O–C_7_H_10_O_2_]^−^, 171.1040[M–H–C_9_H_16_O_2_]^−^	Malyngic acid	CFM-ID
112	10.05	C_16_H_28_O_2_	252.2089	252.2099	3.96	275.2001[M+Na]^+^, 253.2178[M+H]^+^, 219.1756[M+H–H_2_O–CH_3_]^+^, 149.0969[M+H–CH_3_–C_5_H_11_O]^+^	7*δ*-Methoxy-4(14)-oppositen-1*β*-ol	[86]
113 *	10.61	C_15_H_22_O_2_	234.1620	234.1623	1.28	235.1705[M+H]^+^, 177.1273[M+H–H_2_O–C_3_H_4_]^+^, 163.1480[M+H–C_3_H_2_O_2_]^+^, 121.0663[M+H–H_2_O–C_7_H_12_]^+^	Germacra-1(10),4,11(13)-trien-12-oic acid	[87]
114 *	10.65	C_18_H_34_O_5_	330.2406	330.2417	3.33	329.2335[M–H]^−^, 229.1447[M–H–C_6_H_12_O]^−^, 211.1343[M–H–C_6_H_12_O–H_2_O]^−^, 99.0814[M–H–C_12_H_22_O_4_]^−^	9,12,13-TriHOME	CFM-ID
115 *	10.69	C_30_H_32_O_9_	536.2046	536.2063	3.17	535.2021[M–H]^−^, 505.1877[M–H–CH_3_O]^−^, 490.1633[M–H–CH_3_–CH_3_O]^−^	Lappaol A	[88]
116 *	10.96	C_21_H_24_O_6_	372.1573	372.1587	3.76	371.1501[M–H]^−^, 356.1264[M–H–CH_3_]^−^, 136.0528[M–H–C_13_H_15_O_4_]^−^, 121.0094[M–H–C_13_H_15_O_4_–CH_3_]^−^, 83.0144[M–H–C_9_H_11_O_2_–C_8_H_9_O_2_]^−^	Arctigenin	S
117 *	11.14	C_21_H_22_O_6_	370.1416	370.1421	1.35	371.1493[M+H]^+^, 219.0652[M+H–C_9_H_12_O_2_]^+^, 151.0766[M+H–C_12_H_12_O_4_]^+^, 137.0606[M+H–C_13_H_14_O_4_]^+^, 107.0500[M+H–C_13_H_14_O_4_–CH_3_O]^+^	(+)-7,8-Didehydroarctigenin	[89]
118 *	12.10	C_15_H_20_O_2_	232.1463	232.1472	3.88	233.1545[M+H]^+^, 203.1084[M+H–2CH_3_]^+^, 189.1630[M+H–CO_2_]^+^, 149.1335[M+H–C_4_H_4_O_2_]^+^	Costunolide	[87]
119 *	12.94	C_15_H_22_O_2_	234.1620	234.1625	2.14	235.1699[M+H]^+^, 161.1320[M+H–C_3_H_6_O_2_]^+^, 133.1022[M+H–C_5_H_10_O_2_]^+^, 121.1026[M+H–C_6_H_10_O_2_]^+^, 81.0712[M+H–C_9_H_14_O_2_]^+^	Costic acid	[90]
120 *	14.58	C_42_H_46_O_12_	742.2989	742.2978	−1.48	765.2856[M+Na]^+^, 743.3051[M+H]^+^, 725.2928[M+H–H_2_O]^+^, 707.2841[M+H–2H_2_O]^+^, 151.0763[M+H–C_33_H_36_O_10_]^+^, 137.0601[M+H–C_13_H_15_O_4_–C_21_H_23_O_6_]^+^	Diarctigenin	[91]
121 *	15.17	C_42_H_46_O_12_	742.2989	742.2991	0.27	765.2867[M+Na]^+^, 743.3063[M+H]^+^, 725.2951[M+H–H_2_O]^+^, 707.2834[M+H–2H_2_O]^+^, 151.0465[M+H–C_33_H_36_O_10_]^+^, 137.0612[M+H–C_34_H_38_O_10_]^+^	Conicaol A	[91]
122 *	15.41	C_28_H_50_O_2_	418.3811	418.3830	4.54	441.3725[M+Na]^+^, 419.3898[M+H]+, 259.2380[M+H–H_2_O–C_9_H_18_O]^+^, 151.1500[M+H–H_2_O–C_17_H_30_O]^+^, 95.0880[M+H–H_2_O–C_21_H_38_O]^+^	Ergostane-3,24-diol	CFM-ID
123 *	16.06	C_15_H_18_O_2_	230.1307	230.1315	3.48	231.1388[M+H]^+^, 203.1441[M+H–CO]^+^, 121.1028[M+H–C_6_H_6_O_2_]^+^, 105.0718[M+H–C_7_H_10_O_2_]^+^	Dehydrocostus lactone	S
124 *	16.35	C_26_H_48_NO_7_P	517.3168	517.3181	2.51	518.3254[M+H]^+^, 184.0744[M+H– C_21_H_34_O_3_]^+^, 104.1100[M+H–C_21_H_35_O_6_P]^+^, 86.0986[M+H– C_21_H_37_O_7_P]^+^	LPC (18:3)	CFM-ID
125 *	16.40	C_15_H_22_O	218.1671	218.1680	4.13	219.1757[M+H]^+^, 203.1444[M+H–CH_3_]^+^, 162.1419[M+H–C_3_H_5_O]^+^	Germacra-1(10),4,11(13)-trien-12-al	[92]
126 *	16.68	C_18_H_36_O_4_	316.2614	316.2628	4.43	315.2545[M–H]^−^, 297.2453[M–H–H_2_O]^−^, 171.1031[M–H–C_9_H_18_–H_2_O]^−^, 141.1291[M–H–C_9_H_16_O_2_–H_2_O]^−^	9,10-Dihydroxystearic acid	CFM-ID
127 *	16.72	C_16_H_30_O_3_	270.2195	270.222	2.59	293.2116[M+Na]^+^, 269.2124[M+H]^+^, 165.1651[M+H–C_4_H_8_O_3_]^+^, 121.1025[M+H–C_7_H_16_O_3_]^+^, 95.0869[M+H–C_9_H_18_O_3_]^+^	(6Z)-2-Hydroxy-6-hexadecenoic acid	CFM-ID
128 *	16.78	C_18_H_30_O_3_	294.2195	294.2207	4.08	293.2134[M−H]^−^, 275.2035[M−H−H_2_O]^−^, 249.2230[M−H−HCOOH]^−^, 195.1401[M− H–C_6_H_10_O]^−^	13-oxo-9,11-Octadecadienoic acid	CFM-ID
129 *	17.64	C_26_H_50_NO_7_P	519.3325	519.3336	2.12	520.3408[M+H]^+^, 184.0744[M+H– C_21_H_36_O_3_]^+^, 104.1101[M+H–C_21_H_37_O_6_P]^+^, 86.1006[M+H– C_21_H_39_O_7_P]^+^	LPC (18:2)	CFM-ID
130 *	17.92	C_18_H_32_O_3_	296.2351	296.2361	3.38	295.2288[M–H]^−^, 277.2180[M–H_2_O]^−^, 250.2309[M–HCOOH]^−^	Coronaric acid	CFM-ID
131 *	18.46	C_18_H_30_O_3_	294.2195	294.2203	2.72	293.2131[M–H]^−^, 275.2042[M−H−H_2_O]^−^, 249.2230[M−H −HCOOH]^−^, 113.0973[M−H–C_11_H_16_O_2_]^−^	9-Oxo-10,12-Octadecadienoic acid	S
132 *	18.65	C_24_H_50_NO_7_P	495.3325	495.3337	2.42	496.3409[M+H]^+^, 184.0742[M+H– C_19_H_36_O_3_]^+^, 104.1100[M+H– C_19_H_37_O_6_P]^+^, 86.1006[M+H– C_19_H_39_O_7_P]^+^	LPC (16:0)	S
133 *	19.26	C_26_H_52_NO_7_P	521.3481	521.3486	0.96	522.3559[M+H]^+^, 184.0745[M+H– C_21_H_38_O_3_]^+^, 104.1101[M+H– C_21_H_39_O_6_P]^+^, 86.1005[M+H– C_21_H_41_O_7_P]^+^	LPC (18:1)	S
134 *	19.30	C_16_H_22_O_4_	278.1518	278.1526	2.88	301.1419[M+Na]^+^, 279.1571[M+H]^+^, 149.0245[M+H–C_4_H_9_–C_4_H_9_O]^+^, 121.0305[M+H–C_4_H_9_–C_5_H_9_O_2_]^+^	Dibutyl phthalate	[93]
135 *	19.38	C_18_H_34_O_3_	298.2508	298.2519	3.69	297.2446[M–H]^−^,279.2335[M–H–H_2_O]^−^, 253.2542[M−H−HCOOH]^−^	Ricinoleic acid	CFM-ID
136 *	21.00	C_30_H_48_O_4_	472.3553	472.3575	4.66	471.3492[M–H]^−^, 427.3588[M–H–HCOOH]^−^, 411.3273[M–H–HCOOH–CH_3_]^−^	Macrocarpoic acid A	[94]
137 *	22.39	C_18_H_30_O_2_	278.2246	278.2259	4.67	277.2176[M–H]^−^, 259.2076[M–H–H_2_O]^−^, 109.0661[M–H–C_11_H_18_–H_2_O]^−^	Linolenic acid	S
138 *	22.58	C_16_H_32_O_3_	272.2351	272.2386	2.21	271.2314[M–H]^−^, 225.2255[M–H–HCOOH]^−^, 223.2086[M–H–2H_2_O–CH_3_]^−^, 197.1904[M–H–2H_2_O–C_3_H_7_]^−^	3-Hydroxyhexadecanoic acid	CFM-ID
139 *	23.98	C_30_H_48_O	424.3705	424.3700	−1.18	425.3773[M+H]^+^, 205.1942[M+H–C_15_H_24_O]^+^, 189.1644[M+H–C_16_H_28_O]^+^, 161.1335[M+H–C_18_H_32_O]^+^	Lupenone	[95]
140 *	24.04	C_18_H_32_O_2_	280.2402	280.2412	3.57	279.2329[M–H]^−^, 261.2229 [M –H–H_2_O]^−^	Linoleic acid	S
141 *	24.25	C_30_H_48_O	424.3705	424.3695	−2.36	425.3767[M+H]^+^, 409.3454[M+H–CH_3_]^+^, 217.1953[M+H–C_14_H_24_O]^+^, 137.1337[M+H–C_20_H_32_O]^+^	Amyrone	[96]
142 *	24.62	C_30_H_48_O_2_	440.3654	440.3651	−0.68	441.3724[M+H]^+^, 231.2112[M+H– C_13_H_21_O]^+^, 187.1493[M+H–C_16_H_28_O–H_2_O]^+^	Ptiloepoxide	[97]
143 *	24.63	C_30_H_48_O	424.3705	424.3693	−2.83	425.3765[M+H]^+^, 205.1954[M+H– C_15_H_24_O]^+^, 189.1640[M+H– C_16_H_28_O]^+^	Taraxasterone	[98]
144 *	24.82	C_30_H_48_O_2_	440.3654	440.3658	0.91	441.3731[M+H]^+^, 423.3611[M+H–H_2_O]^+^, 191.1803[M+H–C_16_H_26_O_2_]^+^, 123.1191[M+H–C_21_H_34_O_2_]^+^	11*α*-Hydroxyurs-12-en-3-one	[99]
145 *	24.87	C_35_H_36_N_4_O_5_	592.2686	592.2696	1.69	593.2769[M+H]^+^, 533.2556[M+H–C_2_H_4_O_2_]^+^	Pheophorbide A	[100]
146 *	25.44	C_30_H_48_O_2_	440.3654	440.3644	−2.27	441.3717[M+H]^+^, 189.1638[M+H–C_16_H_28_O_2_]^+^, 135.1184 [M+H–C_20_H_34_O_2_]^+^	11-Oxo-amyrin	[101]
147 *	25.60	C_16_H_32_O_2_	256.2402	256.2409	2.73	255.2326[M–H]^−^, 237.2208[M–H−H_2_O]^−^	Hexadecanoic acid	CFM-ID
148 *	27.87	C_28_H_48_O_2_	416.3654	416.3669	3.60	461.3661[M+HCOO]^−^, 415.3591[M–H]^−^, 281.2849[M–H–C_9_H_10_O]^−^	*β*-Tocopherol	CFM-ID
149 *	28.02	C_24_H_38_O_4_	390.2770	390.2770	0.00	413.2662[M+Na]^+^, 391.2847[M+H]^+^, 149.0247[M+H–C_8_H_17_–C_8_H_17_O]^+^	Dioctyl phthalate	[102]

S: compared with the reference compounds. CFM-ID: compared with the CFM-ID 4.0 [103]. * identified from SP for the first time.

**Table 2:** Contents of 20 polyphenols in ethanol extract of SP.

No.	Compound	Regression Equations	*R* ^2^	linearity Range (μg·mL^−1^)	LOD (μg·mL^−1^)	LOQ (μg·mL^−1^)	Content (%)
**2**	Chlorogenic acid	*y* = 35.49*x* + 468.5	0.9991	1~100	0.20	1.0	4.60
**13**	Neochlorogenic acid	*y* = 47.005*x* + 5.7739	0.9993	0.1~10	0.04	0.1	0.13
**60**	Luteolin 7-glucuronide	*y* = 155.56*x* − 19.818	0.9992	0.1~10	0.05	0.1	0.07
**61**	Rutin	*y* = 27.813*x* + 1119	0.9993	1~100	0.33	1.0	6.86
**62**	Isoquercitroside	*y* = 895.09*x* − 133.08	0.9995	0.1~10	0.02	0.1	0.20
**66**	1,4-Dicaffeoylquinic acid	*y* = 174.39*x* + 1766.7	0.9997	1~100	0.33	1.0	2.04
**68**	1,5-Dicaffeoylquinic acid	*y* = 471.27*x* + 438.81	0.9991	0.1~10	0.03	0.1	0.14
**69**	Narcisin	*y* = 56.783*x* + 1673.6	0.9991	1~100	0.20	1.0	6.94
**72**	Syringaresinol	*y* = 879.44*x* − 136.7	0.9992	0.05~5	0.02	0.05	0.006
**73**	Quercitrin	*y* = 3142.4*x* + 151.2	0.9993	0.05~5	0.03	0.05	0.026
**76**	4.5-Dicaffeoylquinic acid	*y* = 522.11*x* + 15.755	0.9994	0.1~10	0.02	0.1	0.52
**78**	Pinoresinol 4-glucoside	*y* = 61.543*x* + 55.902	0.9998	0.1~10	0.05	0.1	0.16
**85**	Matairesinoside	*y* = 702.5*x* − 75.75	0.9993	0.1~10	0.02	0.1	0.08
**93**	Pinoresinol	*y* = 24.76*x* + 1.1	0.9998	1~100	0.20	1.0	1.12
**96**	Arctiin	*y* = 1631*x* + 4406.1	0.9994	0.5~50	0.10	0.5	5.42
**97**	Luteolin	*y* = 3162*x* + 331	0.9996	0.05~5	0.02	0.05	0.01
**102**	Eupafolin	*y* = 17011*x* + 24.231	0.9990	0.05~5	0.02	0.05	0.03
**106**	Apigenin	*y* = 226.54*x* + 5077.6	0.9996	0.5~50	0.20	0.5	4.10
**108**	Matairesinol	*y* = 1519.7*x* + 132.26	0.9997	0.05~5	0.01	0.05	0.01
**116**	Arctigenin	*y* = 39.1*x* − 32.2	0.9995	0.1~10	0.03	0.1	0.76

**Table 3:** The information of identified metabolites in serum and colon.

No.	RT/min	Measured Mass (Da)	Mass Error (ppm)	Adducts	Biomarkers	Sources	Pathway	HMDB ID	Change Trend	
									M/C	D/M
1 *	0.60	132.0300	0.23	M-H	L-Aspartic acid	Colon	ASGM, HD	HMDB0000191	↑	↓
2 ^#^	0.64	124.0069	0.08	M-H	Taurine	Colon	THM	HMDB0000251	↑	↓
3 *	0.68	115.0029	−0.17	M-H	Fumarate	Colon	ASGM, TM, CC	HMDB0000134	↑	↓
4 ^#^	0.79	191.0190	−0.10	M-H	Citrate	Colon	ASGM, CC	HMDB0000094	↑	↓
5 ^#^	0.8	117.0187	−0.09	M-H	Succinate	Serum	CC	HMDB0000254	↓	↑
6 *	0.8	182.0802	−0.82	M+H	L-Tyrosine	Serum	PTTB, TM, PM	HMDB0000158	↓	↑
7 *	0.98	166.0856	−0.72	M+H	L-Phenylalanine	Serum	PTTB, PM	HMDB0000159	↓	↑
8 ^#^	10.51	353.2328	0.00	M-H	Prostaglandin F2a	Colon	AM	HMDB0001139	↑	↓
9 *	10.53	301.2182	0.46	M+H	all-trans-Retinoic acid	Colon	RM	HMDB0001852	↑	↓
10 ^#^	12.81	318.3002	−0.19	M+H	Phytosphingosine	Serum	SM	HMDB0004610	↑	↓
11 ^#^	12.97	300.2907	0.13	M+H	Sphingosine	Colon	SM	HMDB0000252	↑	↓
12 *	13.68	351.2158	−0.37	M-H	Prostaglandin H2	Colon	AM	HMDB0001381	↑	↓
13 *	14.36	335.2222	0.00	M-H	Leukotriene B4	Colon	AM	HMDB0001085	↑	↓
14 ^#^	15.06	302.3052	−0.23	M+H	Sphinganine	Serum	SM	HMDB0000269	↑	↓
15 ^#^	16.33	335.2220	0.00	M-H	5(S)-HpETE	Serum	AM	HMDB0001193	↓	↑
16 *	18.16	319.2272	−0.03	M-H	8,9-EET	Serum	AM	HMDB0002232	↓	↑
17 *	21.69	277.2158	−0.36	M-H	*α*-Linolenic acid	Serum	ALM	HMDB0001388	↑	↓
18 *	22.81	303.2323	−0.03	M-H	Arachidonate	Serum	AM	HMDB0001043	↑	↓
19 *	23.11	279.2324	0.00	M-H	Linoleic acid	Serum	LM	HMDB0000673	↑	↓
20 ^#^	26.60	780.5543	0.00	M+H	PC(18:3/18:2)	Serum	LM, ALM	HMDB0008204	↓	↑
21 ^#^	27.54	756.5532	0.17	M+H	PC(14:0/20:4)	Serum	AM	HMDB0007883	↑	↓

* Metabolites validated with standards. ^#^ Metabolites confirmed by MS data. “↑” represents the content was up-regulated. “↓” represents the content was down-regulated.

**Table 4:** The results from metabolic pathways of differential metabolites.

Pathway Name	Match Status	*p*	−log (*p*)	Holm *p*	FDR	Impact
Phenylalanine, tyrosine and tryptophan biosynthesis (PTTB)	2/4	1.09 × 10^−3^	2.9613	0.0885	0.0230	1.0000
Linoleic acid metabolism (LM)	2/5	1.81 × 10^−3^	2.7431	0.1445	0.0304	1.0000
Arachidonic acid metabolism (AM)	7/36	2.22 × 10^−7^	6.6532	1.87 × 10^−5^	1.87 × 10^−5^	0.5861
Taurine and hypotaurine metabolism (THM)	1/8	0.1065	0.9727	1.0000	0.7141	0.4286
Phenylalanine metabolism (PM)	3/12	4.75 × 10^−4^	3.3234	0.0389	0.0133	0.3571
alpha-Linolenic acid metabolism (ALM)	2/13	0.0132	1.8803	1.0000	0.1230	0.3333
Retinol metabolism (RM)	1/16	0.2021	0.6944	1.0000	0.9433	0.2275
Alanine, aspartate and glutamate metabolism (ASGM)	4/28	4.61 × 10^−4^	3.3361	0.0383	0.0133	0.2260
Sphingolipid metabolism (SM)	3/21	0.0026	2.5772	0.2065	0.0318	0.2028
Tyrosine metabolism (TM)	2/42	0.1142	0.9423	1.0000	0.7141	0.1644
Citrate cycle (TCA cycle) (CC)	3/20	0.0023	2.6403	0.1809	0.0318	0.1529

## Data Availability

The data presented in this study are available on request from the corresponding author.

## Supplementary Materials

The following supporting information can be downloaded at: https://www.mdpi.com/article/10.3390/molecules28041526/s1, Figure S1: The representative BPI chromatograms of serum and colon samples of control, model and SPH groups in negative modes (A-F) and in positive modes (G–L).; Table S1: Precision and accuracy of 20 investigated analytes by UPLC-Q/TOF-MS; Table S2: The RSDs(%)of peak area and RT in validation tests; Table S3: The AUCs and *p* values of the biomarkers in different ROC curves; Table S4. The AUCs and p values of the biomarkers in different ROC curves.

## Sample Availability

Samples of the compounds are available on request from the corresponding author.

## Abbreviations

BPI	base peak intensity
CMC-Na	sodium carboxymethylcellulose
CYN	changyanning tablet
DAI	disease active index
DSS	dextran sodium sulfate
ESI	electron spray ionization
GSH	glutathione
H&E	hematoxylin-eosin staining
IL-6	lnterleukin-6
iNOS	inducible nitric oxide synthase
LOD	limits of detection
LOQ	limits of quantification
LPC	lysophosphatidylcholine
MDA	malondialdehyde
MPO	myeloperoxidase
OPLS-DA	orthogonal projections to latent structures discriminant analysis
PBS	phosphate buffered saline
PCA	principal component analysis
QC	quality control
QTOF-MS	quadrupole time of flight-mass spectrometry
ROC	receiver operating characteristic
RSD	relative standard deviation
RT	retention time
S.E.M.	standard error of the mean
SOD	superoxide dismutase
SP	*Saussurea pulchella*
TEM	transmission electron microscopy
TNF-*α*	tumor necrosis factor-*α*
UC	ulcerative colitis
VIP	variable importance for the projection
