# Supplementary material for: Study on the Comprehensive Phytochemicals and the Anti-Ulcerative Colitis Effect of Saussurea pulchella
Source: Molecules. 2023 Feb 4;28(4):1526. doi: 10.3390/molecules28041526 (PMC9964537; doi:10.3390/molecules28041526)
Supplement: Supplementary file 1 [file molecules-28-01526-s001.zip › molecules-2199073-supplementary.pdf]

Supplementary method

UPLC-Q/TOF-MS

A Waters Acquity UPLC system connected to a Waters Xevo G2-XS QTOF mass spectrometer (Waters Co.,Milford, MA, USA) was used to perform chromatographic separations and mass spectrometry detections via electrospray ionization interface. An ACQUITY UPLC BEH C18 (100 mm × 2.1 mm, 1.7 μm) column with BEH C18 guard column (2.1 mm × 5 mm, 1.7 μm) provided by Waters Corporation was used to perform chromatographic separation. The total flow rate of the mobile phase solution was 0.4 ml·min^{-1} with eluent A (H_2O with 0.1% formic acid) and eluent B (acetonitrile with 0.1% formic acid). The low collision energy and high collision energy of the MS^E mode were 6 V and 20~40 V, respectively. During data acquisition, the data collected by continuum mode. Sodium formate was used to calibrate the mass spectrometer within the scope of 100 to 1200 Da to ensure the mass accuracy and reproducibility. Leucine enkephalin (100 ng·ml^{-1}, *m/z* 554.2615 in ESI- mode and 556.2771 in ESI+ mode) was used as the external reference of Lock Spray™, which was injected at a continuous flow of 10 μL·min^{-1}. The injected volume of samples was 5 μL for each run. The data were collected by a Masslynx™ V4.1 workstation (Waters, Manchester, UK). The temperatures of the autosampler and the column were set at 18 °C and 35 °C, respectively.

For phytochemical analysis, the gradient of qualitative analysis was consisted of 10% B from 0 min to 2 min, 10%→90% B from 2 min to 26 min, 90% B from 26 min to 28 min, back to 10% B from 28 min to 28.1 min, and hold 10% B from 28.1min to 40 min. While the elution procedure program for quantitative analysis was as follows: 10% B from 0 min to 1 min; 10%→90% B from 1 min to 12 min; 90% B from 12 min to 13 min; 90%→10% B in 13.1 min, and 10% B from 13.1 min to 20 min. The weak wash solvent was 10% (v/v) acetonitrile and the strong wash solvent was 90% (v/v) acetonitrile. The optimized MS parameters of both positive mode (ESI+) and negative mode (ESI-) were described as below: capillary voltage 2.2 kV or 2.5 kV; source temperature 150 °C; cone voltage 40 V; cone gas flow 50 L·h^{-1}; desolvation temperature 400 °C; desolvation gas flow 800 L·h^{-1}.

For metabolomic study, the gradient of elution procedure consisted of 0% B (0~2 min); 10%→90% B (2~26 min); 90% B (26~28 min); 90%→10% B (28~28.1 min); 10% B (28.1~40 min). The conditions for the MS system: capillary voltage in positive mode 2.5 kV and in negative mode of 2.3 kV, cone voltage 50 V, source temperature 180 °C, desolvation temperature 350 °C, desolventizing gas flow rate 700 L/h, cone gas flow rate 50 L/h, MSE mode, centroid, low energy 6 V, high energy 20~40 V. QC sample was run randomly four times throughout

the whole worklist. Raw data recording was performed on the MassLynx V4.1 workstation (Waters, Manchester, UK).

Qualitative analysis

Firstly, an independent database was created in addition to the Traditional Medicine Library within the UNIFI platform. Namely, the chemical compositions reported from *Saussurea* species were searched in online databases, including China National Knowledge Infrastructure (CNKI), Web of Science, ChemSpider, Medline and PubMed, and were gathered to form the database including names, chemical structures, and molecular formulas of the components being acquired. Secondly, the MS raw data compressed by Waters Compression and Archival Tool v1.10, were imported into UNIFI software (Waters, Manchester, UK) and were automatically analyzed by the workflow, the main parameters for workflow were as follows: minimum peak area was 200; peak intensities of low and high energy were 200 and 1000 counts, respectively; the acceptable difference of retention time of reference substance was in the range of ±0.1 min. Both positive adducts (+H and +Na) and negative adducts (−H and +COOH) were selected in the analysis. The components matched the evaluation criteria were screened quickly and were listed. Thirdly, the results were refined with a filter (mass error of the molecular weight or the typical fragments in the range of ±5 ppm, response value >5000). Finally, following the above conditions, the compound was identified by comparing retention time and accurate molecular weight with reference substance or by comparing representative MS fragmentation patterns with the literatures.

Metabolomics

Preparation of test samples

In order to make the serum sample free of protein, 150 μL of serum were mixed with 600 μL of pre-cooled methanol. Then, the mixture was kept standing for 10 min at room temperature. Supernatants collected by centrifugation (10,000 rpm, 10 min, 4°C) were lyophilized by vacuum freeze-dryer after being snap-frozen in liquid nitrogen. The dried residue dissolved in 100 μL of methanol/water (4:1) was filtrated with a syringe filter (0.22 μm) to acquire the test sample solution, which could be injected into the UPLC system directly. Additionally, quality control (QC) sample for the method validation was prepared by gathering the same volume (10 μL) from each test sample solution.

According to the above the preparation of the serum test solution, the colon test solution and QC sample were prepared by using 0.1 g of colon tissue homogenized in 80% methanol (1000 mL).

Validation of LC-MS method

Serum or colon QC sample and one serum or colon sample were used to verify the applied method. A total of 10 different mass ions (5 ions from ESI+ and from ESI- modes, respectively) were used to perform the validation test. And the relative standard deviations (RSDs) of peak intensity and retention time (RT, min) of these 10 ions were calculated. The investigation included: the stability of the system being monitored by using QC samples; the precision being evaluated by repeatedly measuring the QC samples for five consecutive times; the reproducibility of sample preparation being evaluated by testing five test solutions that prepared in parallel from the same serum or colon samples; the stability of the post-preparation sample was evaluated by detecting one serum sample or colon sample placed in autosampler for 0, 4, 8 and 12 h at 18 °C.

Analysis method

MarkerLynx XS V4.1 (Waters, Milford, CT, USA) and Simca 15.0 software (Umetrics, Malmo, Sweden) were applied to perform the metabolomic analysis and to visualize the results of the study.

The raw data collected from MarkerLynx were processed by using parameters consisting of mass tolerance 0.10, retention time window 0.10, minimum intensity 20%, marker intensity threshold 5000 counts, and noise elimination level 7. The *m*/*z*-RT pairs with intensities for all screened peaks were displayed in Extend Statistics XS viewer. Then, the results were input into Simca software to carry out principal component analysis (PCA), orthogonal projections to latent structures discriminant analysis (OPLS-DA) and permutation test. The differential metabolites were screened by the variable importance for the projection (VIP) in the OPLS-DA results. Metabolites with FC>2 or <0.5, VIP>1 and $p<0.05$ in volcano map were considered statistically as potential biomarkers. Furthermore, the predictive receiver operating characteristic [1] curves were used to validate the above results, and the area under curve [2] of the ROC curve should be greater than 0.8. HMDB (http://www.hmdb.ca/) was used to identify the metabolites based on the exactly molecular weights and fragmentation patterns. The mass tolerance should be within ±10 ppm. The MetaboAnalyst 4.0 database (http://www.metaboanalyst.ca/) was used to screen potential metabolisms with the impact-value threshold above 0.10.

Network pharmacology

First, to acquire the compounds-related targets, the compounds characterized from SP by qualitative analysis were uploaded to some database, such as the ECTM database (http://www.nrc.ac. cn:9090/ETCM/), TargetNet database (http://targetnet. scbdd.com), SEA database (http://sea.bkslab.org), SwissTargetPrediction database (http://swisstargetprediction.ch/), Symmap database (https://www.bioinfo.org/ symmap), Batman-TCM database (http://bionet.ncpsb.org/batman-tcm/).

Second, with the keywords of "ulcerative colitis", disease-related targets were collected from the following databases: Genecards (https://www.genecards.org/), DisGeNET (https://www.disgenet.org/) and Malacard (https://www.malacards.org/). After merging all the targets and removing duplication, the core targets of compounds and diseases were derived by using the STRING database (https://cn.string-db.org/).

Third, the networks of "compounds in SP-core targets" were constructed by classifying all core targets (enzymes, transporters, receptors, cytokines, proteins, and others). The network was finally generated by topological analysis with Cytoscape software (3.7.1) (http://www. cytoscape.org/) , and the active compounds were screened out by average degrees. All the core targets were considered as the potential therapeutic targets.

Table S1. Compounds identified from SP by UPLC-Q/TOF-MS

NO.	t_R (min)	Formula	Theoretical Mass (Da)	Calculated Mass (Da)	Mass error (ppm)	MS^E Fragmentation	Identification	Ref.
1*	0.71	$C_7H_{12}O_6$	192.0634	192.0643	4.69	191.0570[M–H]$^-$, 173.0443[M–H–H_2O]$^-$	Quinic acid	S
2*	0.79	$C_{16}H_{18}O_9$	354.0951	354.0961	2.82	353.0988[M–H]$^-$, 191.0569[M–H–$C_9H_6O_3$]$^-$, 179.0338[M–H–$C_7H_{10}O_5$]$^-$, 135.0451[M–H–$C_8H_{10}O_7$]$^-$	Chlorogenic acid	S
3*	0.82	$C_6H_{12}O_6$	180.0634	180.0637	1.67	179.0564[M–H]$^-$, 131.0358[M–H–H_2O–CH_2O]$^-$, 103.0404[M–H–H_2O–$C_2H_5O_2$]$^-$	D-Galactose	[3]
4*	0.83	$C_{30}H_{38}O_{15}$	638.2211	638.2232	3.29	683.2214[M–H]$^-$, 489.1377[M–H–Fuc]$^-$, 458.1828[M–H–$C_9H_7O_4$]$^-$, 161.0201[M–H–Glu–Fuc–$C_8H_{11}O_2$]$^-$	Sucrose	[4,5]
5*	0.83	$C_{12}H_{22}O_{11}$	342.1162	342.1179	4.97	341.1106[M–H]$^-$, 179.0571[M–H–Glu]$^-$, 161.0465[M–H–OFru]$^-$	Cistanoside C	[4]
6*	0.94	$C_{10}H_{17}NO_3$	199.1208	199.1213	2.51	200.1286[M+H]$^+$, 168.1017[M+H–CH_3O]$^+$, 126.0930[M+H–C_2H_4–H_2O–CH_3O]$^+$, 122.0978[M+H–H_2O–$C_2H_3O_2$]$^+$, 94.0687[M+H–C_2H_4–H_2O–$C_2H_3O_2$]$^+$	Tussilagine	[1]
7*	0.96	$C_{14}H_{18}O_9$	330.0951	330.0961	3.03	329.0889[M–H]$^-$, 167.0356[M–H–Glu]$^-$	Mudanoside A	[6]
8*	0.98	$C_{13}H_{16}O_{10}$	332.0743	332.0754	3.31	331.0681[M–H]$^-$, 168.0068[M–H–Glu]$^-$, 124.0173[M–H–Glu–CO_2]$^-$	Glucogallin	[7]
9*	1.01	$C_7H_6O_4$	154.0266	154.0264	–1.30	153.0192[M–H]$^-$, 109.0281[M–H–HCOOH]$^-$	Protocatechuic acid	S
10*	1.03	$C_{11}H_{12}O_6$	240.0634	240.0643	3.75	285.0625[M+HCOO]$^-$, 239.0564[M–H]$^-$, 149.0597[M–H–2HCOOH]$^-$, 108.0518[M–H–$C_4H_2O_5$]$^-$	Eucomic acid	[8]
11*	1.08	$C_{15}H_{21}NO_7$	327.1318	327.1323	1.53	328.1395[M+H]$^+$, 310.1288[M+H–H_2O]$^+$, 292.1183[M+H–2H_2O]$^+$, 264.1229[M+H–H_2O–HCOO]$^+$, 166.0867[M+H–$C_6H_{10}O_5$]$^+$	Fructose-phenylalanine	[9]
12*	1.13	$C_{14}H_{20}O_9$	332.1107	332.1121	4.22	331.1049[M–H]$^-$, 168.0431[M–H–Glu]$^-$, 154.0237[M–H–Glu–CH_3]$^-$, 139.0028[M–H–Glu–2CH_3]$^-$, 137.0246[M–H–Glu–CH_3O]$^-$	Leonuriside A	[10]
13*	1.16	$C_{16}H_{18}O_9$	354.0951	354.0964	3.75	353.0887[M–H]$^-$, 191.0568[M–H–$C_9H_6O_3$]$^-$, 135.0456[M–H–$C_8H_{10}O_7$]$^-$	Neochlorogenic acid	S
14*	1.20	$C_{15}H_{18}O_9$	342.0951	342.0965	4.09	341.0892[M–H]$^-$, 179.0342[M–H–Glu]$^-$, 135.0446[M–H–Glu–CO_2]$^-$	Phoeniceoside	[11]
15*	1.24	$C_{15}H_{18}O_8$	326.1002	326.1011	2.76	325.0938[M–H]$^-$, 163.0413[M–H–Glu]$^-$, 119.0513[M–H–Glu–HCOOH]$^-$	Melilotoside	[12]
16*	1.30	$C_{18}H_{18}O_5$	314.1154	314.1162	3.18	315.1235[M+H]$^+$, 193.0875[M+H–$C_7H_6O_2$]$^+$, 147.0451[M+H–CH_3O–$C_8H_9O_2$]$^+$, 137.0622[M+H–$C_{10}H_{10}O_3$]$^+$	*p*-Hydroxyphenethyl ferulate	CFM-ID
17*	1.31	$C_7H_6O_4$	154.0266	154.0270	2.60	153.0197[M–H]$^-$, 109.0293[M–H–HCOOH]$^-$	3,4-Dihydroxybenzoic acid	[13]
18*	1.45	C_9H_7NO	145.0528	145.0523	–3.45	146.0595[M+H]$^+$, 118.0655[M+H–CHO]$^+$	Indole-3-aldehyde	[14]
19*	1.47	$C_{27}H_{28}N_2O_4$	444.2049	444.2035	–3.16	443.1962[M–H]$^-$, 252.1025[M–H–$C_{11}H_{13}O_2N$]$^-$	Cryptochlorogenic acid	S
20*	1.47	$C_{16}H_{18}O_9$	354.0951	354.0962	3.11	353.0889[M–H]$^-$, 307.0824[M–H–HCOOH]$^-$, 191.0566[M–H–$C_9H_6O_3$]$^-$, 146.0587[M–H–$C_9H_6O_3$–HCOOH]$^-$	Aurantiamide acetate	[15]
21*	1.67	$C_{10}H_{12}O_4$	196.0736	196.0742	3.06	241.0724[M+HCOO]$^-$, 195.0661[M–H]$^-$, 179.0721[M–H–H_2O]$^-$, 165.0563[M–H–CH_3O]$^-$	Acetosyringone	[16]
22*	1.76	$C_{18}H_{26}O_9$	386.1577	386.1590	3.37	431.1572[M+HCOO]$^-$, 385.1503[M–H]$^-$, 223.0995[M–H–Glu]$^-$, 135.0467[M–H–OGlu–C_4H_7O]$^-$	Methylsyringin	[17]
23	1.78	$C_{19}H_{27}NO_6$	365.1838	365.1844	1.62	366.1917[M+H]$^+$, 330.1704[M+H–2H_2O]$^+$, 262.1437[M+H–H_2O–$C_4H_7O_2$]$^+$	Pulchellamine B	[18]

24*	1.82	$C_{14}H_{18}O_7$	298.1053	298.1067	4.70	343.1039$[M+HCOO]^-$, 164.0695$[M-H-C_8H_7O_2]^-$, 133.0303$[M-H-Glu]^-$, 121.0300$[M-H-Glu-CH_3]^-$	Ameliaroside	[19]
25*	1.88	$C_{20}H_{27}NO_6$	377.1838	377.1846	2.12	378.1919$[M+H]^+$, 360.1802$[M+H-H_2O]^+$, 332.1862$[M+H-HCOOH]^+$, 314.1749$[M+H-HCOOH-H_2O]^+$, 227.1060$[M+H-2H_2O-C_5H_9NO_2]^+$	Calophyllamine A	[20]
26	1.97	$C_{17}H_{24}O_9$	372.1420	372.1433	3.49	417.1445$[M+HCOO]^-$, 371.1351$[M-H]^-$, 209.0821$[M-H-Glu]^-$, 194.0586$[M-H-CH_3-Glu]^-$, 151.0409$[M-H-Glu-C_3H_5O]^-$	Syringin	[7]
27*	2.08	$C_{20}H_{20}O_8$	388.1158	388.1156	−0.51	411.1048 $[M+Na]^+$, 389.1232$[M+H]^+$, 371.1133$[M+H-H_2O]^+$, 167.0720$[M+H-C_{11}H_{10}O_5]^+$	6*α*-Catechyl-2*α*-guaicyl-3,7-dioxabicyclo[3.3.0]octan-4-one	[21]
28*	2.26	$C_7H_6O_3$	138.0317	138.0323	4.35	137.0251$[M-H]^-$, 109.0302$[M-H-CHO]^-$	Protocatechuic aldehyde	S
29*	2.36	$C_{15}H_{20}O_8$	328.1158	328.1168	3.05	327.1095$[M-H]^-$, 165.0562$[M-H-Glu]^-$, 147.0453$[M-H-H_2O-Glu]^-$	Paeonoside	[22]
30*	2.48	$C_{17}H_{24}O_{10}$	388.1369	388.1387	4.61	387.1305$[M-H]^-$, 371.0989$[M-H-CH_3]^-$, 207.0664$[M-H-OGlu]^-$, 192.0432$[M-H-Glu-CH_3O]^-$	Geniposide	[23]
31*	2.57	$C_{16}H_{18}O_8$	338.1002	338.1010	2.37	337.0931$[M-H]^-$, 191.0562$[M-H-C_9H_7O_2]^-$, 163.0402$[M-H-C_7H_{11}O_5]^-$	3-*p*-Coumaroylquinic acid	[24]
32*	2.61	$C_8H_8O_4$	168.0423	168.0426	1.79	167.0373$[M-H]^-$, 123.0355$[M-H-HCOOH]^-$, 108.0216$[M-H-HCOOH-CH_3]^-$, 93.0343$[M-H-HCOOH-CH_3O]^-$	Vanillic acid	[25]
33*	2.77	$C_9H_{10}O_5$	198.0528	198.0551	1.51	197.0449$[M-H]^-$, 179.0345$[M-H-H_2O]^-$, 135.0444$[M-H-H_2O-HCOOH]^-$	Syringic acid	[26]
34*	2.80	$C_{17}H_{26}O_7$	342.1679	342.1692	3.80	387.1664$[M+HCOO]^-$, 341.1608$[M-H]^-$, 163.1127$[M-H-OGlu]^-$	Jasmolone glucoside	CFM-ID
35*	2.81	$C_9H_8O_4$	180.0423	180.0422	−0.56	179.0340$[M-H]^-$, 135.0438$[M-H-HCOOH]^-$	Caffeic acid	S
36*	2.82	$C_{20}H_{27}NO_6$	377.1838	377.1831	−1.92	378.1904$[M+H]^+$, 332.1854$[M+H-HCOOH]^+$, 257.1408$[M+H-2H_2O-CH_2-C_3H_3O_2]^+$, 235.0971$[M+H-C_3H_3O_2-C_4H_8N]^+$, 206.0939$[M+H-C_3H_3O_2-C_5H_8NO]^+$	Lanicepomine A	[18]
37*	2.98	$C_{13}H_{18}O_6$	270.1103	270.1111	2.96	315.1113$[M+HCOO]^-$, 269.1029$[M-H]^-$, 161.0455$[M-H-C_7H_8O]^-$	Benzyl *β*-D-glucoside	[27]
38*	3.06	$C_{15}H_{16}O_6$	292.0947	292.0958	3.77	337.0930$[M+HCOO]^-$, 291.0873$[M-H]^-$, 163.0414$[M-H-H_2O-C_2HO-C_4H_5O]^-$	Cnidimol D	[28]
39*	3.50	$C_{11}H_{14}O_5$	226.0841	226.0844	1.34	225.0770$[M-H]^-$, 195.0663$[M-H-CH_3O]^-$, 180.0427$[M-H-C_2H_5O]^-$, 149.0240$[M-H-CH_3O-C_2H_5O]^-$	3-Hydroxy-1-(4-hydroxy-3,5-dimethoxyphenyl)propan-1-one	[29]
40*	3.52	$C_{26}H_{34}O_{12}$	538.2050	538.2058	1.49	583.2031$[M+HCOO]^-$, 537.1982$[M-H]^-$, 375.1454$[M-H-Glu]^-$, 357.1342$[M-H-Glu-H_2O]^-$, 151.0407$[M-H-Glu-C_{12}H_{16}O_4]^-$	Medusaside A	[30]
41*	3.58	$C_{17}H_{20}O_9$	368.1107	368.1110	0.81	367.1037$[M-H]^-$, 191.0564$[M-H-CH_3-C_9H_5O_3]^-$, 161.0241$[M-H-C_8H_{14}O_6]^-$, 135.0450$[M-H-C_9H_{12}O_7]^-$	Methyl 3-caffeoylquinate	[31]
42*	3.70	$C_{19}H_{32}O_8$	388.2097	388.2116	4.89	433.2099$[M+HCOO]^-$, 387.2030$[M-H]^-$, 225.1501$[M-H-Glu]^-$, 153.0920$[M-H-C_4H_7O-Glu]^-$	Icariside B8	CFM-ID
43*	3.72	$C_{26}H_{34}O_{12}$	538.2050	538.2068	3.34	583.2039$[M+HCOO]^-$, 537.1980$[M-H]^-$, 375.1451$[M-H-Glu]^-$, 153.0927$[M-H-C_{17}H_{20}O_{10}]^-$	Medusaside B	[30]
44*	3.73	$C_{19}H_{30}O_8$	386.1941	386.1949	2.07	431.1961 $[M+HCOO]^-$, 385.1970$[M-H]^-$, 223.1344$[M-H-Glu]^-$, 205.1231$[M-H-Glu-H_2O]^-$	Saussureoside B	[32]

45	4.21	$C_{20}H_{29}NO_6$	379.1995	379.2002	1.85	380.2075$[M+H]^+$, 334.2013$[M+H-HCOOH]^+$, 316.1910$[M+H-HCOOH-H_2O]^+$, 215.1075$[M+H-2H_2O-C_6H_{11}NO_2]^+$	Pulchellamine E	[18]
46*	4.29	$C_9H_{10}O_3$	166.0630	166.0635	3.01	165.0562$[M-H]^-$, 147.0452$[M-H-H_2O]^-$	Phloretic acid	[33]
47*	4.37	$C_{26}H_{34}O_{12}$	538.2050	538.2069	3.53	537.1986$[M-H]^-$, 375.1451$[M-H-Glu]^-$, 327.1240$[M-H-Glu-H_2O-CH_3O]^-$, 297.1136$[M-H-Glu-H_2O\text{-}2CH_3O]^-$, 225.1250$[M-H-Glu-C_8H_7O_3]$–	Lanicepside A	[34]
48*	4.44	$C_8H_8O_2$	136.0524	136.0527	2.21	135.0455$[M-H]^-$, 120.0213$[M-H-CH_3]^-$, 92.0267$[M-H-C_2H_3O]^-$	Curculigoside C	[35]
49*	4.44	$C_{22}H_{26}O_{12}$	482.1424	482.1439	3.11	481.1346$[M-H]^-$, 197.0455$[M-H-Glu-C_7H_5O_2]^-$, 121.0295$[M-H-Glu-C_9H_9O_5]^-$	*p*-Hydroxyacetophenone	[36]
50*	4.49	$C_{21}H_{34}O_9$	430.2203	430.2220	3.95	429.2127$[M-H]^-$,401.1817$[M-H-C_2H_4]^-$, 267.1603$[M-H-Glu]^-$	4α(15),11β(13)-Tetrahydroridentin B-1 -glucoside	[37]
51*	4.56	$C_{26}H_{34}O_{12}$	538.2050	538.2067	3.16	537.2064$[M-H]^-$, 327.1240$[M-H-Glu-H_2O-CH_3O]^-$, 195.0664$[M-H-Glu-C_{10}H_{11}O_3]^-$, 161.0464$[M-H-C_{20}H_{24}O_7]^-$	Citrusin A	[38]
52*	4.64	$C_{26}H_{34}O_{12}$	538.2050	538.2070	3.72	583.2048$[M+HCOO]^-$, 537.1982$[M-H]^-$, 375.1442$[M-H-Glu]^-$, 327.1245$[M-H-Glu-H_2O-CH_3O]^-$, 179.0561$[M-H-C_{20}H_{22}O_6]^-$	Lanicepside B	[34]
53*	4.66	$C_{32}H_{42}O_{16}$	682.2473	682.2499	3.81	727.2481$[M+HCOO]^-$, 681.2411$[M-H]^-$, 519.1877$[M-H-Glu]^-$, 339.1242$[M-H-2Glu-H_2O]^-$	Pinoresinol diglucoside	S
54*	4.73	$C_{27}H_{36}O_{13}$	568.2156	568.2166	1.76	613.2188$[M+HCOO]^-$, 567.2092$[M-H]^-$, 521.2040$[M-H-H_2O-CH_3O]^-$, 405.1565$[M-H-Glu]^-$, 195.0662$[M-H-Glu-C_{11}H_{13}O_4]^-$	Citrusin B	CFM-ID
55*	5.01	$C_{26}H_{36}O_{11}$	524.2258	524.2240	−3.43	523.2167$[M-H]^-$, 507.1880$[M-H-CH_3]^-$, 361.1690$[M-H-Glu]^-$, 346.1771$[M-H-Glu-CH_3]^-$, 315.1331$[M-H-Glu-CH_3-CH_3O]^-$	(-)-Secoisolariciresinol-4-O-β-D-glucoside	[39]
56*	5.01	$C_{17}H_{20}O_9$	368.1107	368.1116	2.44	367.1033$[M-H]^-$, 179.0346$[M-H-C_8H_{12}O_5]^-$, 161.0247$[M-H-C_8H_{14}O_6]^-$, 135.0472$[M-H-C_9H_{12}O_7]^-$	Methyl 4-caffeoylquinate	[40]
57*	5.08	$C_{19}H_{24}O_8$	380.1471	380.1484	3.42	425.1466$[M+HCOO]^-$, 379.1404$[M-H]^-$, 343.1188$[M-H-2H_2O]^-$	15-Hydroxyjanerin	CFM-ID
58	5.11	$C_{21}H_{31}NO_6$	393.2151	393.2163	3.05	416.2095$[M+Na]^+$, 394.2241$[M+H]^+$, 378.1929$[M+H-CH_3]^+$, 342.1711$[M+H-CH_3-2H_2O]^+$, 262.1448$[M+H-C_6H_{12}O_2-H_2O]^+$, 228.1161$[M+H-C_6H_{12}NO_2-2H_2O]^+$	Pulchellamine G	[18]
59*	5.21	$C_{28}H_{38}O_{13}$	582.2312	582.2303	−1.55	581.2230$[M-H]^-$, 419.1720$[M-H-Glu]^-$, 389.1603$[M-H-Glu-CH_3O]^-$, 373.1298$[M-H-Glu-CH_3-CH_3O]^-$	Lyoniresinol-3α-glucoside	[41]
60*	5.28	$C_{21}H_{18}O_{12}$	462.0798	462.0806	1.73	461.0734$[M-H]^-$, 285.0404$[M-H-Gluac]^-$, 151.0049$[M-H-Gluac\text{-}C_8H_6O_2]^-$, 132.0210$[M-H-Gluac\text{-}C_7H_4O_4]^-$	Luteolin 7-glucuronide	S
61	5.32	$C_{27}H_{30}O_{16}$	610.1534	610.1530	−0.66	609.1457$[M-H]^-$, 461.0731$[M-H-Rha]^-$, 300.0281$[M-H-Glu-Rha]^-$	Rutin	S
62*	5.57	$C_{21}H_{20}O_{12}$	464.0955	464.0978	4.96	463.0906$[M-H]^-$, 300.0280$[M-H-Glu]^-$, 151.0041$[M-H-Glu-C_8H_5O_3]^-$, 150.0328$[M-H-Glu-C_7H_4O_4]^-$	Isoquercitroside	S
63*	5.62	$C_{19}H_{22}O_5$	330.1467	330.1475	2.42	375.1447$[M+HCOO]^-$, 329.1395$[M-H]^-$, 297.1131$[M-H-CH_3-H_2O]^-$, 282.0899$[M-H-CH_3-CH_2-H_2O]^-$, 226.0641$[M-H-H_2O-CH_2-C_4H_7O]^-$	Aguerin B	[42]
64*	5.66	$C_{27}H_{34}O_{12}$	550.2050	550.2069	3.45	595.2042$[M+HCOO]^-$, 549.1984$[M-H]^-$, 519.1876$[M-H-CH_3O]^-$, 387.1454$[M-H-Glu]^-$	Saussurenoside	[43]

65*	5.72	$C_{15}H_{10}O_7$	302.0427	302.0435	2.65	303.0508$[M+H]^+$, 178.0272$[M+H-C_6H_5O_3]^+$, 153.0195$[M+H-C_8H_5O_3]^+$, 108.0216$[M+H-H2O-C_9H_5O_4]^+$	Isoetin	[44]
66*	5.76	$C_{25}H_{24}O_{12}$	516.1268	516.1287	3.68	515.1204$[M-H]^-$, 353.0885$[M-H-C_9H_6O_3]^-$, 335.0776$[M-H-C_9H_9O_4]^-$, 191.0570$[M-H-2C_9H_6O_3]^-$, 179.0353$[M-H-C_{16}H_{16}O_8]^-$	1,4-Dicaffeoylquinic acid	S
67*	5.96	$C_{27}H_{30}O_{15}$	594.1585	594.1598	2.19	593.1515$[M-H]^-$, 285.0407$[M-H-Rut]^-$	Luteolin-7-rutinoside	[45]
68*	6.03	$C_{25}H_{24}O_{12}$	516.1268	516.1271	0.58	515.1198$[M-H]^-$, 353.0878$[M-H-C_9H_6O_3]^-$, 191.0561$[M-H-2C_9H_6O_3]^-$, 179.0352$[M-H-C_{16}H_{16}O_8]^-$	1,5-Dicaffeoylquinic acid	S
69*	6.06	$C_{28}H_{32}O_{16}$	624.1690	624.1720	4.81	623.1647$[M-H]^-$, 351.0735$[M-H-ORha-C_6H_4O_2]^-$, 315.0530$[M-H-Rut]^-$	Narcisin	S
70*	6.15	$C_{27}H_{30}O_{14}$	578.1636	578.1653	2.94	577.1580$[M-H]^-$, 269.0474$[M-H-Neo]^-$	Rhoifolin	[46]
71*	6.18	$C_{21}H_{18}O_{11}$	446.0849	446.0868	4.26	445.0791$[M-H]^-$, 284.0322$[M-H-Glu]^-$, 269.0464$[M-H- OGlu]^-$	Rhein-8-glucoside	[47]
72	6.29	$C_{22}H_{26}O_8$	418.1628	418.1639	2.63	417.1567$[M-H]^-$, 402.1271$[M-H-CH_3]^-$, 387.1080$[M-H-2CH_3]^-$, 181.0521$[M-H-C_{13}H_{11}O_4]^-$	Syringaresinol	S
73	6.29	$C_{21}H_{20}O_{11}$	448.1006	448.1021	3.35	447.0938$[M-H]^-$, 301.0375$[M-H-Rha]^-$, 283.0255$[M-H-Rha-H_2O]^-$, 151.0043$[M-H-Rha-C_8H_5O_3]^-$	Quercitrin	S
74	6.31	$C_{26}H_{32}O_{12}$	536.1894	536.1901	1.31	535.1823$[M-H]^-$, 501.1768$[M-H-2H_2O]^-$, 355.1188$[M-H-Glu-H_2O]^-$, 151.0405$[M-H-Glu-C_{12}H_{13}O_4]^-$	1-Hydroxypinoresinol-1 -glucoside	[48]
75*	6.36	$C_{21}H_{20}O_{10}$	432.1056	432.1075	4.40	477.1057$[M+HCOO]^-$, 431.0993$[M-H]^-$, 285.0405$[M-H-Rha]^-$, 161.0464$[M-H-C_{15}H_{10}O_5]^-$	Afzelin	[49]
76*	6.45	$C_{25}H_{24}O_{12}$	516.1268	516.1292	4.65	515.1219$[M-H]^-$, 353.0891$[M-H-C_9H_6O_3]^-$, 191.0579$[M-H-2C_9H_6O_3]^-$, 179.0359$[M-H-C_{16}H_{16}O_8]^-$	4,5-Dicaffeoylquinic acid	S
77*	6.47	$C_{21}H_{20}O_{10}$	432.1056	432.1071	3.47	431.0988$[M-H]^-$, 269.0461$[M-H-Glu]^-$	Cosmosiin	[49]
78*	6.57	$C_{26}H_{32}O_{11}$	520.1945	520.1931	−2.71	565.1913$[M+HCOO]^-$, 519.1851$[M-H]^-$, 357.1323$[M-H-Glu]^-$, 151.0387$[M-H-Glu-C_{12}H_{13}O_3]^-$	Pinoresinol 4-glucoside	S
79*	6.63	$C_9H_{16}O_4$	188.1049	188.1050	0.53	187.0977$[M-H]^-$, 143.1081$[M-H-HCOOH]^-$, 125.0968$[M-H-H2O-HCOOH]^-$	Azelaic acid	[50]
80*	6.67	$C_{22}H_{22}O_{11}$	462.1162	462.1151	−2.38	461.1078$[M-H]^-$, 446.0853$[M-H-CH_3]^-$, 298.0472$[M-H-Glu]^-$, 283.0244$[M-H-Glu-CH_3]^-$	Thermopsoside	[51]
81*	6.82	$C_{34}H_{30}O_{15}$	678.1585	678.1606	3.10	677.1513$[M-H]^-$, 515.1194$[M-H-C_9H_6O_3]^-$, 497.1098$[M-H-C_9H_8O_4]^-$, 353.0881$[M-H-2C_9H_6O_3]^-$, 179.0346$[M-H-C_{25}H_{22}O_{11}]^-$	1,3,5-Tricaffeoylquinic acid	[52]
82*	6.89	$C_{20}H_{26}O_8$	394.1628	394.1641	3.30	417.1533$[M+Na]^+$, 395.1713$[M+H]^+$, 359.1508$[M+H-2H_2O]^+$, 350.1378$[M+H-C_2H_5O]^+$, 327.1243$[M+H-2H_2O-CH_3O]^+$, 229.0776$[M+H-C_2H_5O-H_2O-C_4H_7O_3]^+$	Methoxyjanerin	[53]
83*	6.93	$C_{20}H_{26}O_6$	362.1729	362.1737	2.21	361.1664$[M-H]^-$, 346.1428$[M-H-CH_3]^-$, 327.1231$[M-H-H_2O-CH_3]^-$, 315.1247$[M-H-CH_3-CH_3O]^-$, 165.0563$[M-H-C_{10}H_{13}O_3-CH_3]^-$	Secoisolariciresinol	S
84*	7.00	$C_{21}H_{22}O_7$	386.1366	386.1361	−1.29	387.1434$[M+H]^+$, 163.0400$[M+H-C_{10}H_{10}O_4-2CH_3]^+$, 135.0453$[M+H-C_{13}H_{16}O_5]^+$	Conicaol B	[54]
85*	7.06	$C_{26}H_{32}O_{11}$	520.1945	520.1959	2.69	519.1876$[M-H]^-$, 357.1345$[M-H-Glu]^-$, 342.1116$[M-H-Glu-CH_3]^-$, 121.0305$[M-H-Glu-C_{13}H_{15}O_4]^-$	Matairesinoside	S
86*	7.09	$C_{20}H_{28}O_7$	380.1835	380.1852	4.47	425.1827$[M+HCOO]^-$, 379.1772$[M-H]^-$, 221.0840$[M-H-C_8H_{14}O_3]^-$, 209.0834$[M-H-C_9H_{14}O_3]^-$	Elemacarmanin	CFM-ID

87*	7.10	$C_{18}H_{22}O_6$	334.1416	334.1421	1.50	357.1353[M+Na]$^+$, 335.1502[M+H]$^+$, 317.1404[M+H–H_2O]$^+$, 137.0614[M+H–H_2O–C_8H_9O–$C_2H_3O_2$]$^+$	7*α*-Hydroxygerin	[55]
88*	7.14	$C_{20}H_{22}O_4$	326.1518	326.1524	1.84	327.1597[M+H]$^+$, 203.1089[M+H–$C_7H_8O_2$]$^+$, 189.0924[M+H–$C_7H_7O_2$–CH_3]$^+$, 137.0614[M+H–$C_{12}H_{14}O_2$]$^+$	Dehydrodiisoeugeno l	[56]
89*	7.28	$C_{22}H_{24}O_8$	416.1471	416.1469	–0.48	417.1542[M+H]$^+$, 399.1435[M+H–H_2O]$^+$, 358.1362[M+H–$C_2H_2O_2$]$^+$, 137.0613[M+H–H_2O–$C_{14}H_{14}O_5$]$^+$	Acetoxypinoresinol	CFM-ID
90*	7.35	$C_{30}H_{34}O_{10}$	554.2152	554.2171	3.43	553.2089[M–H]$^-$, 535.1990[M–H–H_2O]$^-$, 357.1352[M–H–H_2O–$C_{10}H_{10}O_3$]$^-$, 181.0877[M–H–$C_{20}H_{20}O_7$]$^-$	Lappaol E	[57]
91*	7.49	$C_{18}H_{18}O_3$	282.1256	282.1267	3.90	327.1249[M+HCOO]$^-$, 239.0726[M–H–C_3H_6]$^-$, 197.0626[M–H–C_3H_5–C_2H_3–H_2O]$^-$, 163.0405[M–H–C_9H_{10}]$^-$	Obovatol	[58]
92*	7.50	$C_{20}H_{20}O_5$	340.1311	340.1321	2.94	339.1248[M–H]$^-$, 324.1008[M–H–CH_3]$^-$, 293.0825[M–H–CH_3–CH_3O]$^-$, 265.0519[M–H–H_2O–C_4H_8]$^-$	Licocoumarone	[59]
93*	7.51	$C_{20}H_{22}O_6$	358.1416	358.1425	2.51	357.1342[M–H]$^-$, 342.1117[M–H–CH_3]$^-$, 151.0405[M–H–$C_{12}H_{16}O_3$]$^-$, 136.0538[M–H–$C_{12}H_{11}O_3$–H_2O]$^-$	Pinoresinol	S
94*	7.68	$C_{30}H_{34}O_{10}$	554.2152	554.2168	2.89	553.2095[M–H]$^-$,535.1954[M–H–H_2O]$^-$, 517.1888[M–H–2H_2O]$^-$	Lappaol C	[60]
95*	7.72	$C_{17}H_{20}O_4$	288.1362	288.1376	4.86	311.1268[M+Na]$^+$, 289.1457[M+H]+, 230.1312[M+H–$C_2H_3O_2$]$^+$, 202.1370[M+H–$C_2H_3O_2$–CO]$^+$	8*α*-Acetoxydehydrocost uslactone	[61]
96*	8.00	$C_{27}H_{34}O_{11}$	534.2101	534.2118	3.18	579.2090[M+HCOO]$^-$, 533.2035[M–H]$^-$, 371.1512[M–H–Glu]$^-$, 356.1280[M–H–Glu–CH_3]$^-$, 136.0535[M–H–Glu–$C_{13}H_{14}O_4$]$^-$, 121.0306[M–H–Glu–CH_3–$C_{13}H_{14}O_4$]$^-$	Arctiin	S
97*	8.18	$C_{15}H_{10}O_6$	286.0477	286.0488	3.85	285.0415[M–H]$^-$, 151.0044[M–H–$C_8H_6O_2$]$^-$, 133.0308[M–H–$C_7H_4O_4$]$^-$, 107.0144[M–H–$C_9H_6O_4$]$^-$	Luteolin	S
98*	8.23	$C_8H_8O_2$	136.0524	136.0521	–2.21	137.0613[M+H]$^+$, 122.0364[M+H–CH_3]$^+$, 94.0407[M+H–C_2H_3O]$^+$	Phenyl acetate	[62]
99*	8.24	$C_{34}H_{30}O_{15}$	678.1585	678.1614	4.28	677.1521[M–H]$^-$, 515.1210[M–H–$C_9H_6O_3$]$^-$, 353.0895[M–H–2$C_9H_6O_3$]$^-$, 335.0788[M–H–$C_9H_7O_3$–$C_9H_7O_4$]$^-$, 179.0352[M–H–$C_{25}H_{22}O_{11}$]$^-$	3,4,5–Tricaffeoylquinic acid	[63]
100*	8.26	$C_{21}H_{24}O_6$	372.1573	372.1583	2.69	373.1656[M+H]$^+$, 355.1549[M+H–H_2O]$^+$, 137.0617[M+H–$C_{13}H_{16}O_4$]$^+$, 122.0386[M+H–$C_{14}H_{19}O_4$]$^+$	Phillygenin	S
101*	8.46	$C_{30}H_{36}O_9$	540.2359	540.2381	4.07	585.2352[M+HCOO]$^-$, 539.2308[M–H]$^-$, 521.2194[M–H–H_2O]$^-$, 509.2192[M–H–CH_3O]$^-$, 371.1505[M–H–CH_3O–$C_8H_9O_2$]$^-$, 297.1145[M–H–H_2O–$C_{12}H_{16}O_4$]$^-$	Sesquipinsapol B	[64]
102*	8.54	$C_{16}H_{12}O_7$	316.0583	316.0596	4.11	317.0668[M+H]$^+$, 302.0429 [M+H–CH_3]$^+$, 168.0062[M+H–CH_3–$C_8H_6O_2$]$^+$, 140.0506[M+H–$C_9H_5O_4$]$^+$	Eupafolin	S
103*	9.04	$C_{15}H_{24}O_2$	236.1776	236.1786	4.23	237.1858[M+H]$^+$, 219.1771[M+H–H_2O]$^+$, 108.0945[M+H–$C_7H_{13}O_2$]$^+$, 92.0631[M+H–C_3H_7O–H_2O–C_5H_8]$^+$	Eudesma-4(14),11(13)-diene-3*β*,12-diol	[65]
104*	9.08	$C_{31}H_{36}O_{10}$	568.2308	568.2328	3.52	567.2256[M–H]$^-$, 535.1982[M–H–CH_3O]$^-$, 517.1888[M–H–H_2O–CH_3O]$^-$, 191.0714[M–H–$C_{20}H_{24}O_7$]$^-$	Lappaol D	[60]
105*	9.28	$C_{34}H_{37}N_3O_6$	583.2682	583.2684	0.34	584.2757[M+H]$^+$, 438.2385[M+H–$C_9H_6O_2$]$^+$, 292.2026[M+H–2$C_9H_6O_2$]$^+$, 275.1765[M+H–C_9H_6O2–$C_9H_9NO_2$]$^+$, 147.0453[M+H–$C_{25}H_{31}N_3O_4$]$^+$	*N*1,*N*5,*N*10-Tri-*p*-coumaroylspermidin e	[66]

106*	9.33	$C_{15}H_{10}O_5$	270.0528	270.0539	4.07	269.0456[M–H]$^-$, 151.0039[M–H–C_8H_6O]$^-$, 117.0356[M–H–$C_7H_8O_4$]$^-$, 107.0145[M–H–$C_9H_6O_3$]$^-$	Apigenin	S
107	9.38	$C_{26}H_{30}N_2O_6$	466.2104	466.2087	–3.65	489.1989[M+Na]$^+$, 467.2160[M+H]$^+$, 321.1205[M+H–CH_3–C_9H_9N]$^+$, 303.1119[M+H–H_2O–CH_3–C_9H_9N]$^+$, 265.1430[M+H–$C_{11}H_{10}N_2O_2$]$^+$, 202.0747[M+H–$C_{15}H_{21}O_4$]$^+$	Pulchellamine F	[18]
108*	9.47	$C_{20}H_{22}O_6$	358.1416	358.1431	4.19	357.1348[M–H]$^-$, 342.1113[M–H–CH_3]$^-$, 179.0718[M–H–$C_{10}H_{10}O_3$]$^-$, 165.0563[M–H–$C_{10}H_9O_3$–CH_3]$^-$, 122.0370[M–H–$C_{13}H_{15}O_4$]$^-$	Matairesinol	S
109*	9.61	$C_{16}H_{12}O_6$	300.0634	300.0640	2.00	299.0568[M–H]$^-$, 284.0330[M–H–CH_3]$^-$, 256.0384[M–H–C_2H_3O]$^-$, 161.0246[M–H–$C_7H_6O_3$]$^-$	Hispidulin	S
110*	9.71	$C_{18}H_{22}O_5$	318.1467	318.1476	2.83	341.1378[M+Na]$^+$, 319.1556[M+H]$^+$, 287.1297[M+H–CH_3O]$^+$, 189.0917[M+H–C_2H_4O–$C_4H_6O_2$]$^+$	Gerin	[55]
111*	9.81	$C_{18}H_{32}O_5$	328.2250	328.2260	3.05	327.2228[M–H]$^-$, 291.1969[M–H–2H_2O]$^-$, 229.1455[M–H–$C_6H_{10}O$]$^-$, 183.1392[M–H–H_2O–$C_7H_{10}O_2$]$^-$, 171.1040[M–H–$C_9H_{16}O_2$]$^-$	Malyngic acid	CFM-ID
112	10.05	$C_{16}H_{28}O_2$	252.2089	252.2099	3.96	275.2001[M+Na]$^+$, 253.2178[M+H]$^+$, 219.1756[M+H–H_2O–CH_3]$^+$, 149.0969[M+H–CH_3–$C_5H_{11}O$]$^+$	7*δ*-Methoxy-4(14)-oppositen-1*β*-ol	[67]
113*	10.61	$C_{15}H_{22}O_2$	234.1620	234.1623	1.28	235.1705[M+H]$^+$, 177.1273[M+H–H_2O–C_3H_4]$^+$, 163.1480[M+H–$C_3H_2O_2$]$^+$, 121.0663[M+H–H_2O–C_7H_{12}]$^+$	Germacra-1(10),4,11(13)-trien-12-oic acid	[68]
114*	10.65	$C_{18}H_{34}O_5$	330.2406	330.2417	3.33	329.2335[M–H]$^-$, 229.1447[M–H–$C_6H_{12}O$]$^-$, 211.1343[M–H–$C_6H_{12}O$–H_2O]$^-$, 99.0814[M–H–$C_{12}H_{22}O_4$]$^-$	9,12,13-TriHOME	CFM-ID
115*	10.69	$C_{30}H_{32}O_9$	536.2046	536.2063	3.17	535.2021[M–H]$^-$, 505.1877[M–H–CH_3O]$^-$, 490.1633[M–H–CH_3–CH_3O]$^-$	Lappaol A	[69]
116*	10.96	$C_{21}H_{24}O_6$	372.1573	372.1587	3.76	371.1501[M–H]$^-$, 356.1264[M–H–CH_3]$^-$, 136.0528[M–H–$C_{13}H_{15}O_4$]$^-$, 121.0094[M–H–$C_{13}H_{15}O_4$–CH_3]$^-$, 83.0144[M–H–$C_9H_{11}O_2$–$C_8H_9O_2$]$^-$	Arctigenin	S
117*	11.14	$C_{21}H_{22}O_6$	370.1416	370.1421	1.35	371.1493[M+H]$^+$, 219.0652[M+H–$C_9H_{12}O_2$]$^+$, 151.0766[M+H–$C_{12}H_{12}O_4$]$^+$, 137.0606[M+H–$C_{13}H_{14}O_4$]$^+$, 107.0500[M+H–$C_{13}H_{14}O_4$–CH_3O]$^+$	(+)-7,8-Didehydroarctigenin	[70]
118*	12.10	$C_{15}H_{20}O_2$	232.1463	232.1472	3.88	233.1545[M+H]$^+$, 203.1084[M+H–2CH_3]$^+$, 189.1630[M+H–CO_2]$^+$, 149.1335[M+H–$C_4H_4O_2$]$^+$	Costunolide	[68]
119*	12.94	$C_{15}H_{22}O_2$	234.1620	234.1625	2.14	235.1699[M+H]$^+$, 161.1320[M+H–$C_3H_6O_2$]$^+$, 133.1022[M+H–$C_5H_{10}O_2$]$^+$, 121.1026[M+H–$C_6H_{10}O_2$]$^+$, 81.0712[M+H–$C_9H_{14}O_2$]$^+$	Costic acid	[71]
120*	14.58	$C_{42}H_{46}O_{12}$	742.2989	742.2978	–1.48	765.2856[M+Na]$^+$, 743.3051[M+H]$^+$, 725.2928[M+H–H_2O]$^+$, 707.2841[M+H–2H_2O]$^+$, 151.0763[M+H–$C_{33}H_{36}O_{10}$]$^+$, 137.0601[M+H–$C_{13}H_{15}O_4$–$C_{21}H_{23}O_6$]$^+$	Diarctigenin	[72]
121*	15.17	C42H46O12	742.2989	742.2991	0.27	765.2867[M+Na]$^+$, 743.3063[M+H]$^+$, 725.2951[M+H–H_2O]$^+$, 707.2834[M+H–2H_2O]$^+$, 151.0465[M+H–$C_{33}H_{36}O_{10}$]$^+$, 137.0612[M+H–$C_{34}H_{38}O_{10}$]$^+$	Conicaol A	[72]
122*	15.41	$C_{28}H_{50}O_2$	418.3811	418.3830	4.54	441.3725[M+Na]$^+$, 419.3898[M+H]+, 259.2380[M+H–H_2O–$C_9H_{18}O$]$^+$, 151.1500[M+H–H_2O–$C_{17}H_{30}O$]$^+$, 95.0880[M+H–H_2O–$C_{21}H_{38}O$]$^+$	Ergostane-3,24-diol	CFM-ID
123*	16.06	$C_{15}H_{18}O_2$	230.1307	230.1315	3.48	231.1388[M+H]$^+$, 203.1441[M+H–CO]$^+$, 121.1028[M+H–$C_6H_6O_2$]$^+$, 105.0718[M+H–$C_7H_{10}O_2$]$^+$	Dehydrocostus lactone	S

124*	16.35	$C_{26}H_{48}NO_7P$	517.3168	517.3181	2.51	518.3254$[M+H]^+$, 184.0744$[M+H–C_{21}H_{34}O_3]^+$, 104.1100$[M+H–C_{21}H_{35}O_6P]^+$, 86.0986$[M+H– C_{21}H_{37}O_7P]^+$	LPC (18:3)	CFM-ID
125*	16.40	$C_{15}H_{22}O$	218.1671	218.1680	4.13	219.1757$[M+H]^+$, 203.1444$[M+H–CH_3]^+$, 162.1419$[M+H–C_3H_5O]^+$	Germacra-1(10),4,11(13)-trien-12-al	[73]
126*	16.68	$C_{18}H_{36}O_4$	316.2614	316.2628	4.43	315.2545$[M–H]^-$, 297.2453$[M–H–H_2O]^-$, 171.1031$[M–H–C_9H_{18}–H_2O]^-$, 141.1291$[M–H–C_9H_{16}O_2–H_2O]^-$	9,10-Dihydroxystearic acid	CFM-ID
127*	16.72	$C_{16}H_{30}O_3$	270.2195	270.222	2.59	293.2116$[M+Na]^+$, 269.2124$[M+H]^+$, 165.1651$[M+H–C_4H_8O_3]^+$, 121.1025$[M+H–C_7H_{16}O_3]^+$, 95.0869$[M+H–C_9H_{18}O_3]^+$	(6Z)-2-Hydroxy-6-hexadecenoic acid	CFM-ID
128*	16.78	$C_{18}H_{30}O_3$	294.2195	294.2207	4.08	293.2134$[M–H]^-$, 275.2035$[M –H–H_2O]^-$, 249.2230 $[M–H–HCOOH]^-$, 195.1401$[M– H–C_6H_{10}O]^-$	13-oxo-9,11-Octadecadienoic acid	CFM-ID
129*	17.64	$C_{26}H_{50}NO_7P$	519.3325	519.3336	2.12	520.3408$[M+H]^+$, 184.0744$[M+H–C_{21}H_{36}O_3]^+$, 104.1101$[M+H–C_{21}H_{37}O_6P]^+$, 86.1006$[M+H– C_{21}H_{39}O_7P]^+$	LPC (18:2)	CFM-ID
130*	17.92	$C_{18}H_{32}O_3$	296.2351	296.2361	3.38	295.2288$[M–H]^-$, 277.2180$[M–H_2O]^-$, 250.2309$[M–HCOOH]^-$	Coronaric acid	CFM-ID
131*	18.46	$C_{18}H_{30}O_3$	294.2195	294.2203	2.72	293.2131$[M–H]^-$, 275.2042$[M–H–H_2O]^-$, 249.2230$[M–H –HCOOH]^-$, 113.0973$[M–H–C_{11}H_{16}O_2]^-$	9-Oxo-10,12-Octadecadienoic acid	S
132*	18.65	$C_{24}H_{50}NO_7P$	495.3325	495.3337	2.42	496.3409$[M+H]^+$, 184.0742$[M+H–C_{19}H_{36}O_3]^+$, 104.1100$[M+H– C_{19}H_{37}O_6P]^+$, 86.1006$[M+H– C_{19}H_{39}O_7P]^+$	LPC (16:0)	S
133*	19.26	$C_{26}H_{52}NO_7P$	521.3481	521.3486	0.96	522.3559$[M+H]^+$, 184.0745$[M+H–C_{21}H_{38}O_3]^+$, 104.1101$[M+H– C_{21}H_{39}O_6P]^+$, 86.1005$[M+H– C_{21}H_{41}O_7P]^+$	LPC (18:1)	S
134*	19.30	$C_{16}H_{22}O_4$	278.1518	278.1526	2.88	301.1419$[M+Na]^+$, 279.1571$[M+H]^+$, 149.0245$[M+H–C_4H_9–C_4H_9O]^+$, 121.0305$[M+H–C_4H_9–C_5H_9O_2]^+$	Dibutyl phthalate	[74]
135*	19.38	$C_{18}H_{34}O_3$	298.2508	298.2519	3.69	297.2446$[M–H]^-$,279.2335$[M–H–H_2O]^-$, 253.2542$[M–H–HCOOH]^-$	Ricinoleic acid	CFM-ID
136*	21.00	$C_{30}H_{48}O_4$	472.3553	472.3575	4.66	471.3492$[M–H]^-$, 427.3588$[M–H–HCOOH]^-$, 411.3273$[M–H–HCOOH–CH_3]^-$	Macrocarpoic acid A	[75]
137*	22.39	$C_{18}H_{30}O_2$	278.2246	278.2259	4.67	277.2176$[M–H]^-$, 259.2076$[M–H–H_2O]^-$, 109.0661$[M–H–C_{11}H_{18}–H_2O]^-$	Linolenic acid	S
138*	22.58	$C_{16}H_{32}O_3$	272.2351	272.2386	2.21	271.2314$[M–H]^-$, 225.2255$[M–H–HCOOH]^-$, 223.2086$[M–H–2H_2O–CH_3]^-$, 197.1904$[M–H–2H_2O–C_3H_7]^-$	3-Hydroxyhexadecanoic acid	CFM-ID
139*	23.98	$C_{30}H_{48}O$	424.3705	424.3700	-1.18	425.3773$[M+H]^+$, 205.1942$[M+H–C_{15}H_{24}O]^+$, 189.1644$[M+H–C_{16}H_{28}O]^+$, 161.1335$[M+H–C_{18}H_{32}O]^+$	Lupenone	[76]
140*	24.04	$C_{18}H_{32}O_2$	280.2402	280.2412	3.57	279.2329$[M–H]^-$, 261.2229 $[M –H–H_2O]^-$	Linoleic acid	S
141*	24.25	$C_{30}H_{48}O$	424.3705	424.3695	-2.36	425.3767$[M+H]^+$, 409.3454$[M+H–CH_3]^+$, 217.1953$[M+H–C_{14}H_{24}O]^+$, 137.1337$[M+H–C_{20}H_{32}O]^+$	Amyrone	[77]
142*	24.62	$C_{30}H_{48}O_2$	440.3654	440.3651	-0.68	441.3724$[M+H]^+$, 231.2112$[M+H–C_{13}H_{21}O]^+$, 187.1493$[M+H–C_{16}H_{28}O–H_2O]^+$	Ptiloepoxide	[78]
143*	24.63	$C_{30}H_{48}O$	424.3705	424.3693	-2.83	425.3765$[M+H]^+$, 205.1954$[M+H–C_{15}H_{24}O]^+$, 189.1640$[M+H– C_{16}H_{28}O]^+$	Taraxasterone	[79]
144*	24.82	$C_{30}H_{48}O_2$	440.3654	440.3658	0.91	441.3731$[M+H]^+$, 423.3611$[M+H–H_2O]^+$, 191.1803$[M+H–C_{16}H_{26}O_2]^+$, 123.1191$[M+H–C_{21}H_{34}O_2]^+$	11α-Hydroxyurs-12-en-3-one	[80]
145*	24.87	$C_{35}H_{36}N_4O_5$	592.2686	592.2696	1.69	593.2769$[M+H]^+$, 533.2556$[M+H–C_2H_4O_2]^+$	Pheophorbide A	[81]
146*	25.44	$C_{30}H_{48}O_2$	440.3654	440.3644	-2.27	441.3717$[M+H]^+$, 189.1638$[M+H–C_{16}H_{28}O_2]^+$, 135.1184 $[M+H–C_{20}H_{34}O_2]^+$	11-Oxo-amyrin	[82]
147*	25.60	$C_{16}H_{32}O_2$	256.2402	256.2409	2.73	255.2326$[M–H]^-$, 237.2208$[M–H–H_2O]^-$	Hexadecanoic acid	CFM-ID
148*	27.87	$C_{28}H_{48}O_2$	416.3654	416.3669	3.60	461.3661$[M+HCOO]^-$, 415.3591$[M–H]^-$,	β-Tocopherol	CFM-ID

						281.2849[M–H–$C_9H_{10}O$]$^-$		
149*	28.02	$C_{24}H_{38}O_4$	390.2770	390.2770	0.00	413.2662[M+Na]$^+$, 391.2847[M+H]$^+$, 149.0247[M+H–C_8H_{17}–$C_8H_{17}O$]$^+$	Dioctyl phthalate	[83]

S: compared with the reference compounds.

CFM-ID: compared with the CFM-ID 4.0 [84].

* identified from SP for the first time

Table S2. Precision and accuracy of 20 investigated analytes by UPLC-Q/TOF-MS

No.	Compound	Precision (RSD, %)		Accuracy (n=5)	
		intra- (*n*=5)	inter- (*n*=6)	Mean	RSD (%)
**2**	Chlorogenic acid	2.49	1.34	98.8	0.90
**13**	Neochlorogenic acid	2.42	2.34	101.5	2.43
**60**	Luteolin 7-glucuronide	2.60	2.44	99.9	2.68
**61**	Rutin	2.57	2.77	101.5	2.29
**62**	Isoquercitroside	2.70	2.84	96.8	2.28
**66**	1,4-Dicaffeoylquinic acid	1.74	1.41	100.4	0.99
**68**	1,5-Dicaffeoylquinic acid	2.38	2.99	98.2	2.79
**69**	Narcisin	1.94	2.55	99.0	0.94
**72**	Syringaresinol	2.35	1.32	96.4	2.52
**73**	Quercitrin	1.48	1.95	100.4	2.67
**76**	4.5-Dicaffeoylquinic acid	1.96	2.49	101.0	1.87
**78**	Pinoresinol 4- glucoside	1.85	2.12	99.7	2.34
**85**	Matairesinoside	2.42	2.91	98.9	2.08
**93**	Pinoresinol	1.32	2.89	98.8	1.70
**96**	Arctiin	2.10	2.90	97.9	1.82
**97**	Luteolin	2.78	1.83	98.5	1.52
**102**	Eupafolin	2.85	1.84	96.4	2.01
**106**	Apigenin	2.12	2.59	100.8	1.04
**108**	Matairesinol	1.91	2.31	98.7	2.44
**116**	Arctigenin	2.49	2.10	98.2	1.97

Table S3. The RSDs（%）of peak area and RT in validation tests

Tests	ESI- mode		ESI+ mode	
	Peak area	RT	Peak area	RT
System stability	1.20~2.92	0.25~2.31	1.13~2.17	0.18~2.56
Precision	0.47~2.65	0.10~2.20	1.01~2.75	0.26~2.04
Reproducibility	1.68~2.92	0.12~2.84	1.01~2.75	0.13~2.38
Sample stability	0.95~2.55	0.28~2.36	0.91~2.66	0.52~2.08

Figure S1. The representative BPI chromatograms of serum and colon samples of control, model and SPH groups in negative modes (A-F) and in positive modes (G–L).

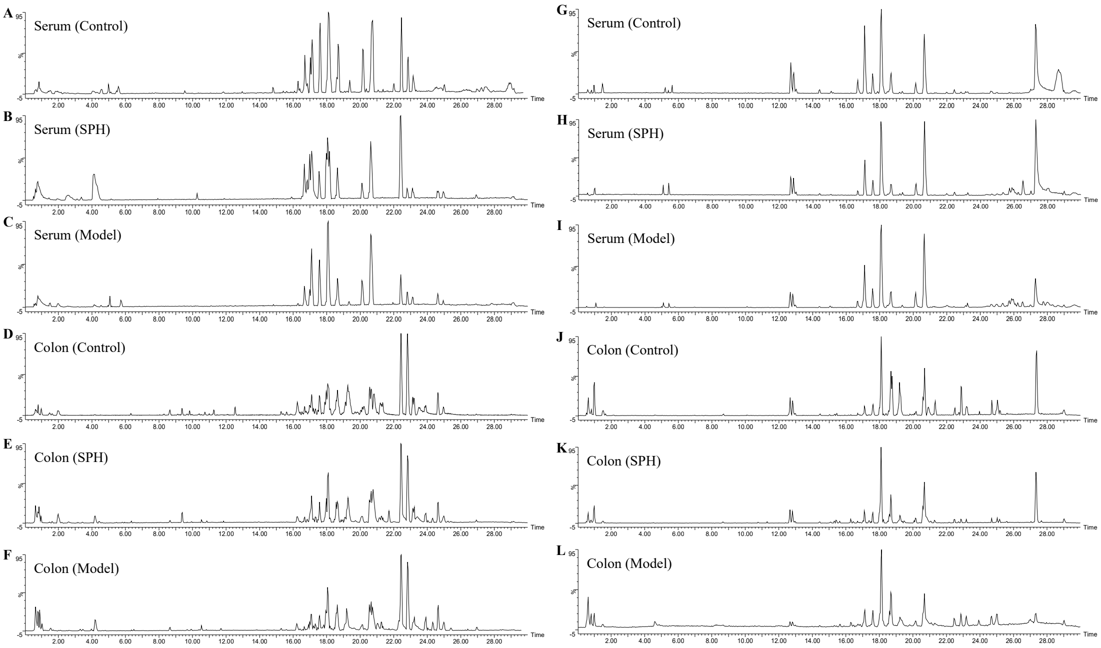

Table S4. The AUCs and p values of the biomarkers in different ROC curves

HMDB No.	Control & Model		SPH & Model	
	AUC	*p*	AUC	*p*
HMDB0000094	1.000	<0.001	1.000	<0.001
HMDB0000134	1.000	<0.001	1.000	<0.001
HMDB0000158	0.990	<0.001	0.910	0.01
HMDB0000159	0.920	<0.001	0.900	0.001
HMDB0000191	1.000	<0.001	1.000	<0.001
HMDB0000251	1.000	<0.001	1.000	<0.001
HMDB0000252	0.980	0.01	0.855	0.01
HMDB0000254	1.000	<0.001	1.000	0.01
HMDB0000269	1.000	<0.001	0.990	0.01
HMDB0000673	1.000	<0.001	1.000	0.01
HMDB0001043	1.000	<0.001	1.000	<0.001
HMDB0001085	1.000	<0.001	0.990	0.01
HMDB0001139	0.990	<0.001	0.900	0.01
HMDB0001193	1.000	<0.001	0.930	<0.001
HMDB0001381	1.000	<0.001	1.000	0.001
HMDB0001388	1.000	<0.001	1.000	<0.001
HMDB0001852	0.910	<0.001	0.900	0.01
HMDB0002232	1.000	<0.001	1.000	<0.001
HMDB0004610	1.000	<0.001	1.000	<0.001
HMDB0007883	1.000	<0.001	0.960	<0.001
HMDB0008204	1.000	<0.001	1.000	<0.001

References:

1. Smyrska-Wieleba, N.; Wojtanowski, K.K.; Mroczek, T. Comparative HILIC/ESI-QTOF-MS and HPTLC studies of pyrrolizidine alkaloids in flowers of Tussilago farfara and roots of Arnebia euchroma. *Phytochemistry Letters* **2017**, *20*, 339-349.
2. Yamauchi, Y.; Ge, Y.-W.; Yoshimatsu, K.; Komatsu, K.; Kuboyama, T.; Yang, X.; Tohda, C. Memory Enhancement by Oral Administration of Extract of Eleutherococcus senticosus Leaves and Active Compounds Transferred in the Brain. *Nutrients* **2019**, *11*, 1142.
3. Korul'Kina, L.; Shul'ts, E.; Zhusupova, G.; Abilov, Z.A.; Erzhanov, K.; Chaudri, M. Biologically active compounds from Limonium gmelinii and L. popovii I. *Chem. Nat. Compd.* **2004**, *40*, 465-471.
4. Yang, Z.-Y.; Lu, D.-Y.; Yao, S.; Zhang, R.-R.; Jiang, Z.-J.; Ma, Z.-G. Chemical fingerprint and quantitative analysis of Cistanchedeserticola by HPLC-DAD-ESI-MS. *J. Food Drug Anal.* **2013**, *21*, 50-57.
5. Zhang, K.; Liu, W.; Song, Q.; Wan, J.-B.; Yu, J.; Gong, X.; Cao, L.; Si, D.; Tu, P.; Li, J. Integrated Strategy Drives Direct Infusion–Tandem Mass Spectrometry as an Eligible Tool for Shotgun Pseudo-Targeted Metabolomics of Medicinal Plants. *Anal. Chem.* **2021**, *93*, 2541-2550.
6. He, J.; Dong, Y.; Liu, X.; Wan, Y.; Gu, T.; Zhou, X.; Liu, M. Comparison of chemical compositions, antioxidant, and anti-photoaging activities of Paeonia suffruticosa flowers at different flowering stages. *Antioxidants* **2019**, *8*, 345.
7. Kumar, S.; Chandra, P.; Bajpai, V.; Singh, A.; Srivastava, M.; Mishra, D.; Kumar, B. Rapid qualitative and quantitative analysis of bioactive compounds from Phyllanthus amarus using LC/MS/MS techniques. *Industrial Crops and Products* **2015**, *69*, 143-152.
8. Llorach, R.; Favari, C.; Alonso, D.; Garcia-Aloy, M.; Andres-Lacueva, C.; Urpi-Sarda, M. Comparative metabolite fingerprinting of legumes using LC-MS-based untargeted metabolomics. *Food Res. Int.* **2019**, *126*, 108666.
9. Hau, J.; Devaud, S.; Blank, I. Detection of Amadori compounds by capillary electrophoresis coupled to tandem mass spectrometry. *Electrophoresis* **2004**, *25*, 2077-2083.
10. Otsuka, H.; Takeuchi, M.; Inoshiri, S.; Sato, T.; Yamasaki, K. Phenolic compounds from Coix lachryma-jobi var. ma-yuen. *Phytochemistry* **1989**, *28*, 883-886.
11. Lin, L.-Z.; Sun, J.; Chen, P.; Harnly, J. UHPLC-PDA-ESI/HRMS/MS n analysis of anthocyanins, flavonol glycosides, and hydroxycinnamic acid derivatives in red mustard greens (Brassica juncea Coss variety). *J. Agric. Food Chem.* **2011**, *59*, 12059-12072.
12. Yang, Y.-L.; Al-Mahdy, D.A.; Wu, M.-L.; Zheng, X.-T.; Piao, X.-H.; Chen, A.-L.; Wang, S.-M.; Yang, Q.; Ge, Y.-W. LC-MS-based identification and antioxidant evaluation of small molecules from the cinnamon oil extraction waste. *Food Chem.* **2022**, *366*, 130576.
13. Bartsch, M.; Bednarek, P.; Vivancos, P.D.; Schneider, B.; von Roepenack-Lahaye, E.; Foyer, C.H.; Kombrink, E.; Scheel, D.; Parker, J.E. Accumulation of isochorismate-derived 2, 3-dihydroxybenzoic 3-O-β-D-xyloside in Arabidopsis resistance to pathogens and ageing of leaves. *J. Biol. Chem.* **2010**, *285*, 25654-25665.
14. Mujahid, M.; Sasikala, C.; Ramana, C.V. Aniline-induced tryptophan production and identification of indole derivatives from three purple bacteria. *Curr. Microbiol.* **2010**, *61*, 285-290.
15. Shakya, R.; Navarre, D.A. Rapid screening of ascorbic acid, glycoalkaloids, and phenolics in potato using high-performance liquid chromatography. *J. Agric. Food Chem.* **2006**, *54*, 5253-5260.

16. Liu, L.; Cui, Z.-x.; Zhang, Y.-b.; Xu, W.; Yang, X.-w.; Zhong, L.-j.; Zhang, P.; Gong, Y. Identification and quantification analysis of the chemical constituents from Mahonia fortune using Q-Exactive HF Mass Spectrometer and UPLC–ESI-MS/MS. *J. Pharm. Biomed. Anal.* **2021**, *196*, 113903.
17. Antunes, A.C.; Acunha, T.d.S.; Perin, E.C.; Rombaldi, C.V.; Galli, V.; Chaves, F.C. Untargeted metabolomics of strawberry (Fragaria x ananassa 'Camarosa') fruit from plants grown under osmotic stress conditions. *J. Sci. Food Agric.* **2019**, *99*, 6973-6980.
18. Yang, M.C.; Choi, S.U.; Choi, W.S.; Kim, S.Y.; Lee, K.R. Guaiane sesquiterpene lactones and amino acid-sesquiterpene lactone conjugates from the aerial parts of Saussurea pulchella. *J. Nat. Prod.* **2008**, *71*, 678-683.
19. Kammerer, B.; Kahlich, R.; Biegert, C.; Gleiter, C.H.; Heide, L. HPLC-MS/MS analysis of willow bark extracts contained in pharmaceutical preparations. *Phytochemical Analysis: An International Journal of Plant Chemical and Biochemical Techniques* **2005**, *16*, 470-478.
20. Milutinović, V.; Niketić, M.; Krunić, A.; Nikolić, D.; Petković, M.; Ušjak, L.; Petrović, S. Sesquiterpene lactones from the methanol extracts of twenty-eight Hieracium species from the Balkan Peninsula and their chemosystematic significance. *Phytochemistry* **2018**, *154*, 19-30.
21. Duan, H.; Takaishi, Y.; Momota, H.; Ohmoto, Y.; Taki, T. Immunosuppressive constituents from Saussurea medusa. *Phytochemistry* **2002**, *59*, 85-90.
22. Xu, S.j.; Yang, L.; Zeng, X.; Zhang, M.; Wang, Z.t. Characterization of compounds in the Chinese herbal drug Mu-Dan-Pi by liquid chromatography coupled to electrospray ionization mass spectrometry. *Rapid Communications in Mass Spectrometry: An International Journal Devoted to the Rapid Dissemination of Up-to-the-Minute Research in Mass Spectrometry* **2006**, *20*, 3275-3288.
23. Saravanakumar, K.; Park, S.; Sathiyaseelan, A.; Kim, K.-N.; Cho, S.-H.; Mariadoss, A.V.A.; Wang, M.-H. Metabolite profiling of methanolic extract of Gardenia jaminoides by LC-MS/MS and GC-MS and its anti-diabetic, and anti-oxidant activities. *Pharmaceuticals* **2021**, *14*, 102.
24. Anttonen, M.J.; Karjalainen, R.O. High-performance liquid chromatography analysis of black currant (Ribes nigrum L.) fruit phenolics grown either conventionally or organically. *J. Agric. Food Chem.* **2006**, *54*, 7530-7538.
25. Fang, N.; Yu, S.; Prior, R.L. LC/MS/MS characterization of phenolic constituents in dried plums. *J. Agric. Food Chem.* **2002**, *50*, 3579-3585.
26. Owen, R.; Haubner, R.; Hull, W.; Erben, G.; Spiegelhalder, B.; Bartsch, H.; Haber, B. Isolation and structure elucidation of the major individual polyphenols in carob fibre. *Food Chem. Toxicol.* **2003**, *41*, 1727-1738.
27. Tolonen, A.; Hohtola, A.; Jalonen, J. Comparison of electrospray ionization and atmospheric pressure chemical ionization techniques in the analysis of the main constituents from Rhodiola rosea extracts by liquid chromatography/mass spectrometry. *J. Mass Spectrom.* **2003**, *38*, 845-853.
28. Hu, H.; Yau, L.-F.; Peng, J.; Hu, B.; Li, J.; Li, Y.; Huang, H. Comparative Research of Chemical Profiling in Different Parts of Fissistigma oldhamii by Ultra-High-Performance Liquid Chromatography Coupled with Hybrid Quadrupole-Orbitrap Mass Spectrometry. *Molecules* **2021**, *26*, 960.

29. Duan, L.; Xiong, H.; Du, Y.; Wang, Z.; Li, Y.; Zhao, S.; Chen, J.; Si, D.; Pan, H. High-throughput LC–MS method for the rapid characterisation and comparative analysis of multiple ingredients of four hawthorn leaf extracts. *Phytochem. Anal.* **2022**.
30. Fan, C.-Q.; Yue, J.-M. Biologically active phenols from Saussurea medusa. *Biorg. Med. Chem.* **2003**, *11*, 703-708.
31. Jaiswal, R.; Kuhnert, N. How to identify and discriminate between the methyl quinates of chlorogenic acids by liquid chromatography–tandem mass spectrometry. *J. Mass Spectrom.* **2011**, *46*, 269-281.
32. Xie, H.; Wang, T.; Matsuda, H.; Morikawa, T.; Yoshikawa, M.; Tani, T. Bioactive constituents from Chinese natural medicines. XV. Inhibitory effect on aldose reductase and structures of saussureosides A and B from Saussurea medusa. *Chem. Pharm. Bull.* **2005**, *53*, 1416-1422.
33. Knust, U.; Erben, G.; Spiegelhalder, B.; Bartsch, H.; Owen, R.W. Identification and quantitation of phenolic compounds in faecal matrix by capillary gas chromatography and nano-electrospray mass spectrometry. *Rapid Communications in Mass Spectrometry: An International Journal Devoted to the Rapid Dissemination of Up-to-the-Minute Research in Mass Spectrometry* **2006**, *20*, 3119-3129.
34. Zhou, Z.W.; Yin, S.; Wang, X.N.; Fan, C.Q.; Li, H.; Yue, J.M. Two new lignan glycosides from Saussurea laniceps. *Helv. Chim. Acta* **2007**, *90*, 951-956.
35. Mellegård, H.; Stalheim, T.; Hormazabal, V.; Granum, P.; Hardy, S. Antibacterial activity of sphagnum acid and other phenolic compounds found in Sphagnum papillosum against food-borne bacteria. *Lett. Appl. Microbiol.* **2009**, *49*, 85-90.
36. Wu, D.; Wang, H.; Tan, J.; Wang, C.; Lin, H.; Zhu, H.; Liu, J.; Li, P.; Yin, J. Pharmacokinetic and metabolism studies of curculigoside C by UPLC-MS/MS and UPLC-QTOF-MS. *Molecules* **2018**, *24*, 21.
37. Yang, D.S.; Whang, W.K.; Kim, I.H. The constituents of Taraxacum hallaisanensis roots. *Arch. Pharmacal Res.* **1996**, *19*, 507-513.
38. Rodríguez-Pérez, C.; Quirantes-Piné, R.; Fernández-Gutiérrez, A.; Segura-Carretero, A. Comparative characterization of phenolic and other polar compounds in Spanish melon cultivars by using high-performance liquid chromatography coupled to electrospray ionization quadrupole-time of flight mass spectrometry. *Food Res. Int.* **2013**, *54*, 1519-1527.
39. LIANG, Y.-H. Lignans and flavonoids from rhizome of Drynaria fortunei. *Chinese Traditional and Herbal Drugs* **2011**, 25-30.
40. Michalska, A.; Wojdyło, A.; Bogucka, B. The influence of nitrogen and potassium fertilisation on the content of polyphenolic compounds and antioxidant capacity of coloured potato. *J. Food Compost. Anal.* **2016**, *47*, 69-75.
41. Cao, Y.; Gu, C.; Zhao, F.; Tang, Y.; Cui, X.; Shi, L.; Xu, L.; Yin, L. Therapeutic effects of Cyathula officinalis Kuan and its active fraction on acute blood stasis rat model and identification constituents by HPLC-QTOF/MS/MS. *Pharmacogn. Mag.* **2017**, *13*, 693.
42. Ha, T.J.; Jang, D.S.; Lee, J.R.; Lee, K.D.; Lee, J.; Hwang, S.W.; Jung, H.J.; Nam, S.H.; Park, K.H.; Yang, M.S. Cytotoxic effects of sesquiterpene lactones from the flowers ofHemisteptia lyrata B. *Arch. Pharmacal Res.* **2003**, *26*, 925-928.
43. Kuo, Y.-H.; Way, S.-T.; Wu, C.-H. A new triterpene and a new lignan from Saussurea japonica. *J. Nat. Prod.* **1996**, *59*, 622-624.

44. Yang, N.; Wang, H.; Lin, H.; Liu, J.; Zhou, B.; Chen, X.; Wang, C.; Liu, J.; Li, P. Comprehensive metabolomics analysis based on UPLC-Q/TOF-MS E and the anti-COPD effect of different parts of Celastrus orbiculatus Thunb. *RSC Advances* **2020**, *10*, 8396-8420.
45. Han, Y.; Zhou, M.; Wang, L.; Ying, X.; Peng, J.; Jiang, M.; Bai, G.; Luo, G. Comparative evaluation of different cultivars of Flos Chrysanthemi by an anti-inflammatory-based NF-κB reporter gene assay coupled to UPLC-Q/TOF MS with PCA and ANN. *J. Ethnopharmacol.* **2015**, *174*, 387-395.
46. Gattuso, G.; Caristi, C.; Gargiulli, C.; Bellocco, E.; Toscano, G.; Leuzzi, U. Flavonoid glycosides in bergamot juice (Citrus bergamia Risso). *J. Agric. Food Chem.* **2006**, *54*, 3929-3935.
47. Ye, M.; Han, J.; Chen, H.; Zheng, J.; Guo, D. Analysis of phenolic compounds in rhubarbs using liquid chromatography coupled with electrospray ionization mass spectrometry. *J. Am. Soc. Mass Spectrom.* **2007**, *18*, 82-91.
48. Wei, L.; Mei, Y.; Zou, L.; Chen, J.; Tan, M.; Wang, C.; Cai, Z.; Lin, L.; Chai, C.; Yin, S. Distribution patterns for bioactive constituents in pericarp, stalk and seed of Forsythiae fructus. *Molecules* **2020**, *25*, 340.
49. Dai, X.; Zhuang, J.; Wu, Y.; Wang, P.; Zhao, G.; Liu, Y.; Jiang, X.; Gao, L.; Xia, T. Identification of a flavonoid glucosyltransferase involved in 7-OH site glycosylation in tea plants (Camellia sinensis). *Sci. Rep.* **2017**, *7*, 1-14.
50. Flores, R.M.; Doskey, P.V. Evaluation of multistep derivatization methods for identification and quantification of oxygenated species in organic aerosol. *J. Chromatogr.* **2015**, *1418*, 1-11.
51. Fu, S.; Arráez-Roman, D.; Segura-Carretero, A.; Menéndez, J.A.; Menéndez-Gutiérrez, M.P.; Micol, V.; Fernández-Gutiérrez, A. Qualitative screening of phenolic compounds in olive leaf extracts by hyphenated liquid chromatography and preliminary evaluation of cytotoxic activity against human breast cancer cells. *Anal. Bioanal. Chem.* **2010**, *397*, 643-654.
52. Lin, L.-Z.; Harnly, J.M. Identification of hydroxycinnamoylquinic acids of arnica flowers and burdock roots using a standardized LC-DAD-ESI/MS profiling method. *J. Agric. Food Chem.* **2008**, *56*, 10105-10114.
53. Singh, P.; Bhala, M. Guaianolides from Saussurea candicans. *Phytochemistry* **1988**, *27*, 1203-1205.
54. Fan, C.-Q.; Zhu, X.-Z.; Zhan, Z.-J.; Ji, X.-Q.; Li, H.; Yue, J.-M. Lignans from Saussurea conica and their NO production suppressing activity. *Planta Med.* **2006**, *72*, 590-595.
55. Wang, X.R.; Wu, Q.X.; Shi, Y.P. Terpenoids and sterols from Saussurea cauloptera. *Chem. Biodivers.* **2008**, *5*, 279-289.
56. Zhang, Y.-B.; Yang, X.-B.; Yang, X.-W.; Xu, W.; Li, F.; Gonzezal, F.J. Liquid chromatography with tandem mass spectrometry: A sensitive method for the determination of dehydrodiisoeugenol in rat cerebral nuclei. *Molecules* **2016**, *21*, 321.
57. Ichihara, A.; Numata, Y.; Kanai, S.; Sakamura, S. New sesquilignans from Arctium lappa L. The structure of lappaol C, D and E. *Agric. Biol. Chem.* **1977**, *41*, 1813-1814.
58. Joo, J.; Lee, D.; Wu, Z.; Shin, J.H.; Lee, H.S.; Kwon, B.M.; Huh, T.L.; Kim, Y.W.; Lee, S.J.; Kim, T.W. In vitro metabolism of obovatol and its effect on cytochrome P450 enzyme activities in human liver microsomes. *Biopharm. Drug Disposition* **2013**, *34*, 195-202.
59. Freund, D.M.; Martin, A.C.; Cohen, J.D.; Hegeman, A.D. Direct detection of surface localized specialized metabolites from Glycyrrhiza lepidota (American licorice) by leaf spray mass spectrometry. *Planta* **2018**, *247*, 267-275.

60. Aabideen, Z.U.; Mumtaz, M.W.; Akhtar, M.T.; Mukhtar, H.; Raza, S.A.; Touqeer, T.; Saari, N. Anti-obesity attributes; UHPLC-QTOF-MS/MS-based metabolite profiling and molecular docking insights of Taraxacum officinale. *Molecules* **2020**, *25*, 4935.
61. Bohlmann, F.; Singh, P.; Jakupovic, J.; Huneck, S. Further guaianolides from Saussurea species. *Planta Med.* **1985**, *51*, 74-75.
62. Ratnam, K.J.; Reddy, R.S.; Sekhar, N.; Kantam, M.L.; Figueras, F. Sulphated zirconia catalyzed acylation of phenols, alcohols and amines under solvent free conditions. *J. Mol. Catal. A: Chem.* **2007**, *276*, 230-234.
63. Zheleva-Dimitrova, D.; Gevrenova, R.; Zaharieva, M.M.; Najdenski, H.; Ruseva, S.; Lozanov, V.; Balabanova, V.; Yagi, S.; Momekov, G.; Mitev, V. HPLC-UV and LC–MS analyses of acylquinic acids in Geigeria alata (DC) Oliv. & Hiern. and their contribution to antioxidant and antimicrobial capacity. *Phytochem. Anal.* **2017**, *28*, 176-184.
64. Barrero, A.F.; Haidour, A.; Dorado, M.M. Sesquipinsapols A and B: two sesquilignans from Abies pinsapo. *Nat. Prod. Lett.* **1993**, *2*, 255-262.
65. Fan, C.Q.; Zhan, Z.J.; Li, H.; Yue, J.M. Eudesmane-Type Sesquiterpene Derivatives from Saussurea conica. *Helv. Chim. Acta* **2004**, *87*, 1446-1451.
66. Lee, J.H.; Lee, S.J.; Park, S.; Kim, H.K.; Jeong, W.Y.; Choi, J.Y.; Sung, N.-J.; Lee, W.S.; Lim, C.-S.; Kim, G.-S. Characterisation of flavonoids in Orostachys japonicus A. Berger using HPLC–MS/MS: Contribution to the overall antioxidant effect. *Food Chem.* **2011**, *124*, 1627-1633.
67. Choi, S.U.; Yang, M.C.; Lee, K.H.; Kim, K.H.; Lee, K.R. Lignan and terpene constituents from the aerial parts of Saussurea pulchella. *Arch. Pharmacal Res.* **2007**, *30*, 1067-1074.
68. Liu, Q.; Majdi, M.; Cankar, K.; Goedbloed, M.; Charnikhova, T.; Verstappen, F.W.; De Vos, R.C.; Beekwilder, J.; Van der Krol, S.; Bouwmeester, H.J. Reconstitution of the costunolide biosynthetic pathway in yeast and Nicotiana benthamiana. *PLoS One* **2011**, *6*, e23255.
69. Aboutabl, E.A.; El Mahdy, M.E.; Sokkar, N.M.; Sleem, A.A.; Shams, M.M. Bioactive lignans and other phenolics from the roots, leaves and seeds of Arctium lappa L. grown in Egypt. *Egyptian Pharmaceutical Journal* **2012**, *11*, 59.
70. Matsumoto, T.; Hosono-Nishiyama, K.; Yamada, H. Antiproliferative and apoptotic effects of butyrolactone lignans from Arctium lappa on leukemic cells. *Planta Med.* **2006**, *72*, 276-278.
71. Nguyen, D.T.; Göpfert, J.C.; Ikezawa, N.; MacNevin, G.; Kathiresan, M.; Conrad, J.; Spring, O.; Ro, D.-K. Biochemical conservation and evolution of germacrene A oxidase in Asteraceae. *J. Biol. Chem.* **2010**, *285*, 16588-16598.
72. Huh, J.; Lee, C.-M.; Lee, S.; Kim, S.; Cho, N.; Cho, Y.-C. Comprehensive Characterization of Lignans from Forsythia viridissima by UHPLC-ESI-QTOF-MS, and Their NO Inhibitory Effects on RAW 264.7 Cells. *Molecules* **2019**, *24*, 2649.
73. de Kraker, J.-W.; Franssen, M.C.; de Groot, A.; Shibata, T.; Bouwmeester, H.J. Germacrenes from fresh costus roots. *Phytochemistry* **2001**, *58*, 481-487.
74. Roy, R.N.; Laskar, S.; Sen, S. Dibutyl phthalate, the bioactive compound produced by Streptomyces albidoflavus 321.2. *Microbiol. Res.* **2006**, *161*, 121-126.
75. Piacente, S.; Santos, L.C.D.; Mahmood, N.; Pizza, C. Triterpenes from Maytenus macrocarpa and evaluation of their anti-HIV activity. *Nat. Prod. Commun.* **2006**, *1*, 1934578X0600101201.

76. Na, M.; Kim, B.Y.; Osada, H.; Ahn, J.S. Inhibition of protein tyrosine phosphatase 1B by lupeol and lupenone isolated from Sorbus commixta. *J. Enzyme Inhib. Med. Chem.* **2009**, *24*, 1056-1059.
77. Frankenberger, L.; D. Mora, T.; de Siqueira, C.D.; Filippin-Monteiro, F.B.; de Moraes, M.H.; Biavatti, M.W.; Steindel, M.; Sandjo, L.P. UPLC-ESI-QTOF-MS2 characterisation of Cola nitida resin fractions with inhibitory effects on NO and TNF-α released by LPS-activated J774 macrophage and on Trypanosoma cruzi and Leishmania amazonensis. *Phytochem. Anal.* **2018**, *29*, 577-589.
78. Menichini, F.; Di Benedetto, R.; Delle Monache, F. A triterpene epoxide and a guaianolide from Ptilostemmon gnaphaloides. *Phytochemistry* **1996**, *41*, 1377-1379.
79. Pütter, K.M.; van Deenen, N.; Müller, B.; Fuchs, L.; Vorwerk, K.; Unland, K.; Bröker, J.N.; Scherer, E.; Huber, C.; Eisenreich, W. The enzymes OSC1 and CYP716A263 produce a high variety of triterpenoids in the latex of Taraxacum koksaghyz. *Sci. Rep.* **2019**, *9*, 1-13.
80. Luis, J.G.; Andrés, L.S. New ursane type triterpenes from Salvia mellifera greene. *Nat. Prod. Lett.* **1999**, *13*, 187-194.
81. Pop, R.M.; Weesepoel, Y.; Socaciu, C.; Pintea, A.; Vincken, J.-P.; Gruppen, H. Carotenoid composition of berries and leaves from six Romanian sea buckthorn (Hippophae rhamnoides L.) varieties. *Food Chem.* **2014**, *147*, 1-9.
82. Rontani, J.-F.; Charrière, B.; Menniti, C.; Aubert, D.; Aubert, C. EIMS Fragmentation and MRM quantification of autoxidation products of α-and β-amyrins in natural samples. *Rapid Communications in Mass Spectrometry: RCM* **2018**.
83. Takatori, S.; Kitagawa, Y.; Kitagawa, M.; Nakazawa, H.; Hori, S. Determination of di (2-ethylhexyl) phthalate and mono (2-ethylhexyl) phthalate in human serum using liquid chromatography-tandem mass spectrometry. *J. Chromatogr. B* **2004**, *804*, 397-401.
84. Wang, F.; Dana, A.; Tian, S.; Eponine, O.; Vasuk, G.; Russell, G.; Thomaso, M.; Davids, W. CFM-ID 4.0 – a web server for accurate MS-based metabolite identification. *Nucleic Acids Res.* **2022**, W1.
